# Differential regulation of mesoscale chromosome conformations in osteoblasts and osteosarcoma

**DOI:** 10.1186/s13059-025-03785-2

**Published:** 2025-09-26

**Authors:** Madhoolika Bisht, Yu-Chieh Chung, Siou-Luan He, Sydney Willey, Benjamin D. Sunkel, Meng Wang, Benjamin Z. Stanton, Li-Chun Tu

**Affiliations:** 1 Department of Biological Chemistry and Pharmacology, The Ohio State University, Columbus, OH 43210 USA; 2 Department of Molecular Genetics, The Ohio State University, Columbus, OH 43210 USA; 3 Center for RNA Biology, The Ohio State University, Columbus, OH 43210 USA; 4 The Ohio State University Comprehensive Cancer Center, The Ohio State University, Columbus, OH 43210 USA; 5 Center for Childhood Cancer, Nationwide Children’s Hospital, Columbus, OH 43205 USA; 6 Department of Pediatrics, The Ohio State University College of Medicine, Columbus, OH 43210 USA; 7 Present Address: Neuroscience Graduate Program, University of Michigan, Ann Arbor, MI 48109 USA

**Keywords:** Chromosome conformation, Live-cell imaging, Osteosarcoma, CRISPR, Chromosome dynamics

## Abstract

**Background:**

Chromosome conformation within the nucleus is essential for genome function. These have primarily been studied at the scale of loops and compartments, or at lower spatial resolution using traditional in situ hybridization in chemically fixed samples. However, the mesoscale organization of single chromosomes in vivo, shaped by the interplay between chromatin architectural proteins and histone modifications, remains partially understood. In this study, we interrogated the mesoscale conformations of interphase chromosomes in live human osteoblasts and transformed osteosarcoma cells, focusing on chromosome 19.

**Results:**

Chromosome conformations were quantified by the aspect ratio of the principal axes of gyration tensors. In osteoblasts, approximately 81% of chromosome 19 are observed to consist of regions characterized by highly extended organizations, with aspect ratios approximately four times greater than those of spheres. In contrast, in osteosarcoma cells, the chromosome displays an extensively collapsed conformation, with aspect ratios more closely approximately that of a sphere. In both cell types, the chromosome’s conformation is bimodal and the balance between these two modes differs very significantly between the two cell types. While the mesoscopic conformation is considerably stable, it is superimposed on dynamic, smaller scale regions. Additional results reveal that this significant conformational shift is independent of the cell cycle but co-regulated by CTCF, cohesion, and H3K27 modifications.

**Conclusions:**

Our findings provide new insights into the coordinated complex regulatory mechanisms governing mesoscale chromosome organization in normal and transformed osteogenic tissues.

**Supplementary Information:**

The online version contains supplementary material available at 10.1186/s13059-025-03785-2.

## Background

Three-dimensional (3D) chromatin architecture is dynamically organized in the cell nucleus to regulate DNA accessibility, essential for cellular processes like transcription and replication [1, 2]. The nucleosome, where DNA wraps around histone octamers, is the fundamental unit of chromatin [3]. Despite its role in DNA packaging, nucleosomes are highly dynamic and can unwrap through entropic processes and ATP-dependent remodeling [4]. Linker histones and posttranslational modifications (PTMs) on histone tails, such as methylation and acetylation [5], further modulate nucleosome spacing and local electrostatic environments [6], influencing gene expression by recruiting transcriptional cofactors like remodelers and silencers [7]. Dysregulation of histone PTMs has been reported in various cancers [8, 9].

Histone modifications, such as methylation of histone H3 at lysine-4 (H3K4me3) and acetylation of histone H3 at lysine-27 (H3K27ac), are associated with euchromatin and essential gene expression [10]. The combination of these histone modifications mainly occurs at active promoters and enhancers [11]. Conversely, repressive histone modifications, such as methylation of histone H3 at lysine-9 (H3K9me2/3) and lysine-27 (H3K27me3), are often found in heterochromatin and involved in gene repression [6]. Each histone modification is added, removed, and read by specific enzymes. In the case of H3K27me3, a methyl group is added by the polycomb repressive complexes (PRC) [12] and removed by the demethylases UTX and JMJD3 [13].

Although chromatin loops can be established in the absence of chromatin architectural proteins [14, 15], chromosome conformation capture-based techniques have revealed topologically associating domains (TADs) [16, 17] regulated by the chromatin architectural proteins, CCCTC binding factor (CTCF), and cohesin [18, 19]. There is evidence that TADs, with a median size of 360 kb in length, extrude through cohesin and are anchored by two convergent CTCF molecules at loop boundaries [20, 21]. Groups of loops form compartments, typically ranging from 1 to 10 megabases, with compartment A being gene-rich and marked by euchromatin marks, and compartment B being gene-poor and commonly marked by heterochromatin marks [22]. Compartments are thought to be dynamic and heterogeneous among cells [21, 23]. To date, compartments have been visualized in fixed cells of specific types but their biological importance is not clear [24, 25]. Thus, segregation and conversion between compartments in human cells remain largely enigmatic. Each interphase chromosome contains several compartments and occupies a distinct 3D nuclear space known as chromosome territory, which limits interchromosomal interactions, thereby maintaining genome integrity [26].

Aberrant 3D genome organization is implicated in cancer. Studies have linked changes in chromatin architecture with tumorigenesis, such as altered *TP53* expression in prostate cancer [27] and disrupted promoter-enhancer loops in breast cancer [23]. Analysis of somatic mutations from ~ 42 different human cancer types showed that changes in the cancer genome largely occurred at TAD boundaries, indicating a strong association with changes in spatial genome organization [28, 29].

Recent advances in high-throughput sequencing and fluorescence in-situ hybridization (FISH) imaging techniques have greatly built our knowledge on 3D genome organization in high resolution, though these techniques require fixed samples, limiting insights into dynamic genome organization beyond size and shape. The mesoscale subcellular structure has been defined as cellular complexes ranging from 100 nm to 10 µm [30]. In this work, we use “mesoscale” to refer specifically to large chromatin domains that range approximately from 2 to 10 µm, which corresponds to the typical size of a human chromosome territory. Chromatin dynamics across the cell nucleus have been investigated primarily using fluorescent histones (e.g., H2B-GFP, etc.), providing insights into nucleosome densities and coherent chromatin movements. It remains unclear whether mesoscopic domains of a single chromosome form static, open structure across all cell types [31]. Gene expression has been shown to drive changes in local chromatin organization [32–34]. In mice, chromatin topologies are cell-type dependent, suggesting an integration of lineage specification and architectural signature [35]. Despite being highly challenging, the temporal evolution of chromosome organization at single chromosome level in live single cells is critical for elucidating the interplay of genome function and structure in detail.

Live-cell imaging techniques such as CRISPRainbow and CRISPR-Sirius [36–38] have provided new insights into dynamic chromatin organization. Early live-cell imaging techniques were focused on specific regions like telomeres and centromeres [39], but whole-genome labeling, such as fluorescent histones, has revealed the dynamics of chromatin architecture [40]. However, these techniques lack gene and chromosome specificity. Insertion of the bacterial lac operon allows local imaging at specific genomic loci but potential effects from foreign DNA bound by exogenous proteins, such as interactions with PML bodies, were reported [41, 42]. CRISPR imaging has been used to study chromatin dynamics on endogenous genomic sequences, including telomeres, pericentromeric regions, active versus silenced genes, and TADs’ boundaries [43–45]. The high precision of CRISPR had been used to visualize aneuploidy and chromosome translocation in cancer cells [46]. In this study, we utilized CRISPR-Sirius to investigate chromatin conformation at a mesoscale (> 10 Mb) in osteosarcoma (OS) and osteoblast (OB) cells. We observed striking differences in 3D conformations of chromosome 19 between osteoblasts and OS. While extended chromosome conformations had previously been described in other species, this is the first report of such conformations in human osteoblast cells [47]. In this work, we quantified mesoscale chromosome conformations in live human cells and uncovered the factors that regulate the conformations, which has not been characterized before. Our findings suggest that chromatin regulators controlling osteogenic 3D conformations operate independently, with limited cross-talk. Additionally, these mesoscale chromosome conformations are stable for hours but exhibit local fluctuation on smaller length scales, reflecting a potential broader phenomenon of gene-rich chromosome dynamics in higher eukaryotes.

## Results

### Chromosome 19 long-arm conformations and gene profiles are distinct in osteoblasts and osteosarcoma

**Fig. 1:**
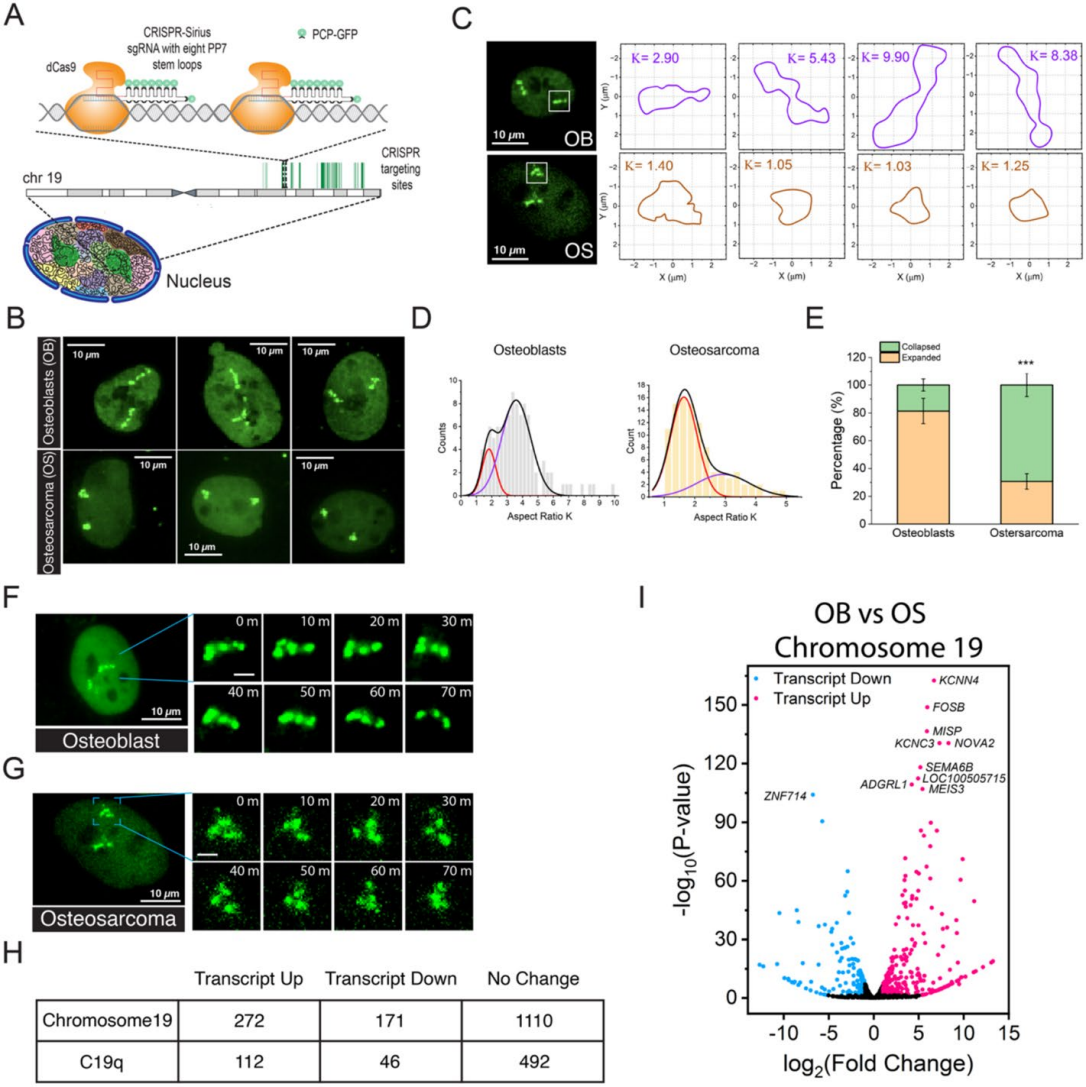
Distinct chromosome conformations in osteoblasts and osteosarcoma cells. **A** A schematic of CRISPR-Sirius. The CRISPR-Sirius single guide RNA (sgRNA) is engineered with an octet of PP7 hairpins (8XPP7). PP7 is labeled by binding to its coat protein (PCP) fused with the green fluorescent protein (GFP). The sgRNA targeted about 836 repeats spanning 17 Mb region on chromosome 19 q arm (C19q), which allows the visualization of chromosome 19 territory. **B** Images of C19q conformation in osteoblasts (OB, hFOB1.19) and osteosarcoma (OS, U2OS) cells. Osteoblasts have extended C19q conformations, whereas osteosarcoma cells demonstrated collapsed C19q conformations. **C** Schematic of C19q conformation quantitative analysis. The spline curves from C19q images in Fig. 1B and 1C were used to calculate gyration tensors that give rise to the aspect ratio (K) of C19q conformations in osteoblasts (upper row) and osteosarcoma cells (lower row). **D** Gaussian bimodal fitting of aspect ratio (K) distribution of C19q conformations for osteoblast (N_C19q_= 104) and osteosarcoma cells (N_C19q_=100). The black lines represent fittings with two Gaussian peaks for whole distributions. Red and purple lines represent the individual distributions for collapsed and extended conformations, respectively. **E** The percentages of collapsed and extended conformations from gaussian bimodal fittings. Histogram shows that osteosarcoma cells (N_C19q_=100) (***, *p* < 0.001) have significantly fewer C19q in the extended conformation as compared to those in osteoblasts (N_C19q_= 104). **F** Time lapse of C19q over 70 minutes in an osteoblast cell. **G** Time lapse of C19q over 70 minutes in an osteosarcoma cell. The m denotes minutes. Bar in the enlarged inset, 2 µm. Each Z-stack was 3D deconvoluted and projected using maximal intensity. **H** Up- and down-regulated on chromosome 19 and specifically on the C19q in osteosarcoma cells, relative to osteoblasts. No change means silenced genes remain silenced, and active genes are expressed at similar levels (log2 fold change is between −1 and 1, calculated by DESeq2). **I** Volcano plot of RNA-seq comparing differential expression of chromosome 19 genes in OB and OS (log2 fold change >1, *p*<0.05). The significance test in **E** was calculated using Fisher’s exact test. All experiments were repeated at least three times, except for 1H and 1I (data from two replicates)

We used CRISPR-Sirius to visualize and investigate mesoscale chromosome conformations in living cells [37]. In this system, crRNA and tracrRNA were fused into a single guide RNA (sgRNA) scaffold engineered with eight PP7 aptamers (8xPP7) (Fig. 1A). The green fluorescent protein fused PP7 coat protein (PCP-GFP) was used for visualization and the nuclease-dead CRISPR-associated protein 9 (dCas9), with mutations at D10A and H840A, was employed to prevent endonuclease activity, which can generate unwanted DNA double stranded breaks [48]. These plasmids were previously described in earlier work [36, 37].

We focused on Human chromosome 19 (chr19) for this study due to its high gene density—more than twice the genome-wide average[49]—and its association with cancer progression and poor prognosis[50–55]. Using CRISPR-Sirius, we targeted ~ 836 repetitive sequences on the long arm (C19q) spanning ~ 17 Mb between 19q13.2 and 19q13.42 (Fig. 1A) [56]. C19q was fluorescently labeled in stable hFOB1.19 cells (OB), and U2OS osteosarcoma (OS) cells, generated via lentiviral transductions (Fig. 1B). The region targeted by C19q was previously verified in U2OS cells using FISH oligopaint [56].

3D imaging revealed distinct C19q conformations between OB and OS cells, with OB cells primarily exhibiting extended and OS cells mostly showing collapsed conformations (Fig. 1B). To quantify C19q conformations, we calculated C19q aspect ratio *K*, a dimensionless parameter derived from the principal axes of the gyration tensor, used to characterize polymer shapes [57, 58]. The aspect ratio *K* of C19q is given by the ratio of eigenvalues of the gyration tensor calculated from a spline curve of C19q (Fig. 1C; see “Materials and methods” section). The smaller value, the rounder conformation. *K* value of 1 corresponds to a round shape, and higher values of *K* indicate more extended conformations. The distribution of *K* values was fitted into Gaussian bimodal distributions (Fig. 1D). For OS cells, the mean *K* values for collapsed and extended C19q are 1.65 and 2.93, respectively, while for OB cells, the mean *K* values are 1.82 (collapsed) and 3.58 (extended). Approximately 81% of C19q conformations in OB cells were extended, compared to 31% in OS cells (Fig. 1E, total number of cells, n ≥ 100, ***p < 0.001). C19q was predominantly in collapsed conformations in OS cells (69%) (Fig. 1E).

To examine conformational stability, we tracked temporal conformational changes by using 3D time-lapse images. We found that the extended C19q in osteoblasts remains extended for 70 min (Movie S1 and Fig. 1F). Similarly, the collapsed C19q in OS cells remained collapsed but underwent significant subdomain fluctuations within 10 min (Movie S2 and Fig. 1G). The C19q conformations were stable for up to 5 h (data not shown). To rule out cell-cycle stage effects, we synchronized both cell lines to the G1 phase using a double thymidine block followed by nocodazole treatment [44, 59, 60]. C19q conformations were captured 6 h after nocodazole release, which corresponds to the mid-late G1 phase. C19q conformations were similar in synchronized and asynchronized cells (Additional file 1: Fig. S1), suggesting that the observed conformational differences were not due to cell-cycle stage but reflect the ability of 3D chromatin architecture to reorganize in disease conditions such as OS.

We also explored whether the distinct C19q conformations correlate with gene expression profiles. RNA-seq analysis revealed that 443 chr19 genes (29%) were differentially expressed in OS cells, with 158 (36%) located within the C19q region (Fig. 1H and I). Whole genome gene set enrichment analysis (GSEA) in oncology indicated that genes associated with immune response, quality control of damaged cells, osteogenic tissue identities, and abnormal tissue metabolism were dysregulated in OS. OS cells exhibited reduced response to virus and radiation, suggesting a weakened immune system and radiotherapy resistance. Additionally, OS cells showed diminished osteoclast development and decreased expression of genes related to bone mineralization, regulation of cell adhesion, and bone development, which may contribute to the bone fragility observed in osteosarcoma patients.

### Transcription inhibition alone has no detectable effects on C19q chromosome conformations

**Fig. 2:**
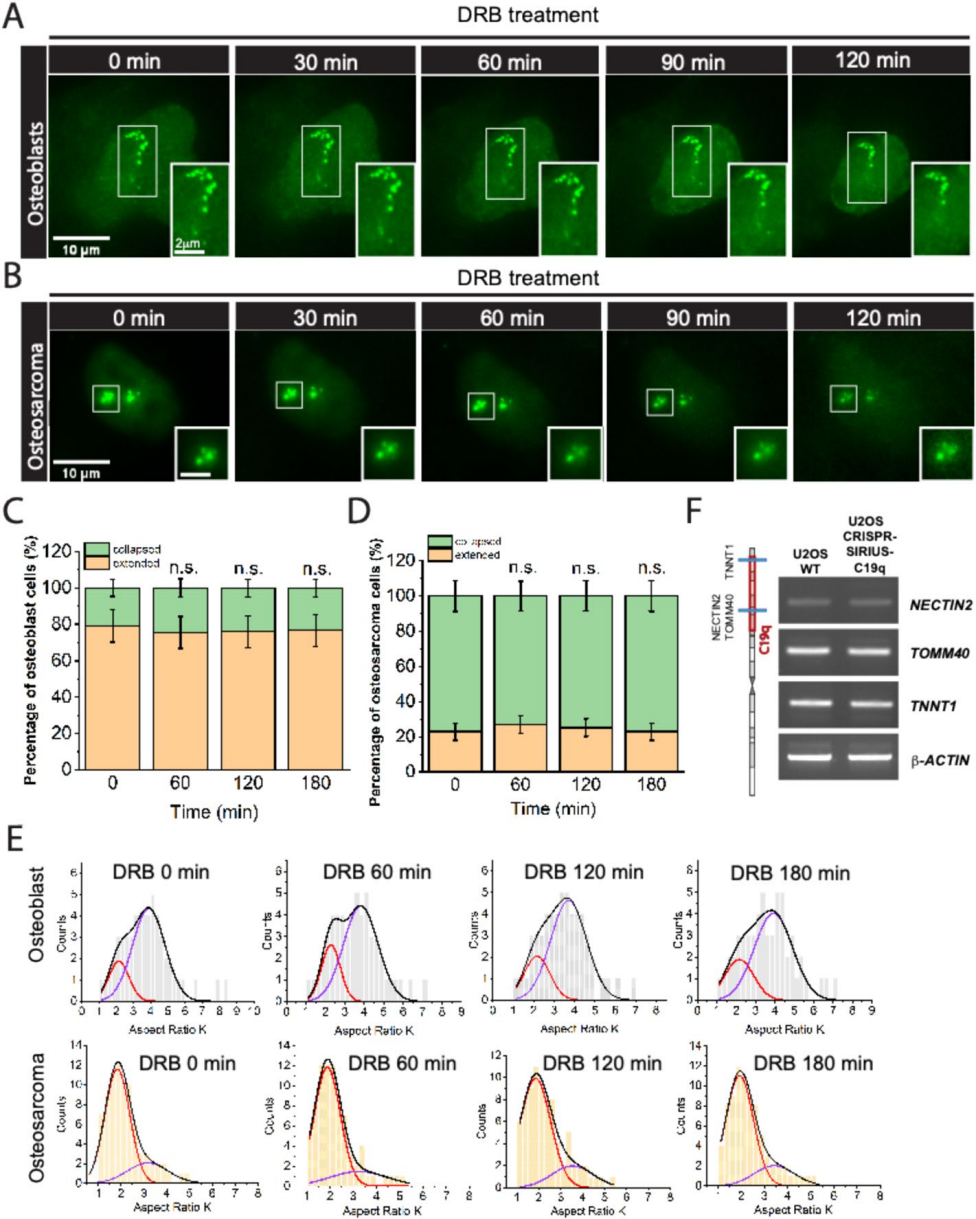
Transcription inhibition has no detectable effects on mesoscale chromosome conformation. **A** Time-lapse images of chromosome conformation under DRB treatment for 120 minutes in osteoblast cells. Transcription inhibition by DRB treatment does not change the extended chromosome conformation. **B** Time-lapse images of chromosome conformation under DRB treatment for 120 minutes in osteosarcoma cells. Transcription inhibition does not affect the collapsed chromosome conformation in osteosarcoma cells. **C** Percentages of osteoblast cells with extended and collapsed C19q conformation were measured from Gaussian bimodal fittings in (**E**) at 60 minutes (N_C19q_=55, *p*= 0.8228), 120 minutes (N_C19q_=55, *p*-value = ~1), and 180 minutes (N_C19q_=55, *p*-value = ~1) (n.s, non-significant). **D** Percentages of osteosarcoma cells with extended and collapsed C19q conformation were measured from Gaussian bimodal fittings in (**E**) at 60 minutes (N_C19q_=80, *p*-value = ~1), 120 minutes (N_C19q_=80, *p*-value = ~1), and 180 minutes (N_C19q_=80, *p*-value = ~1). n.s., non-significant. The significance test was calculated by using Fisher’s exact test. **E** Distributions of aspect ratio K of OB and OS in various DRB treatment conditions are indicated on the graphs. The black lines represent fittings with two Gaussian peaks for the whole distributions. Red and purple lines represent the individual distributions for collapsed and extended conformations, respectively. All experiments were repeated at least three times. **F** RT-PCR of gene transcripts in C19q in OS cells with and without CRISPR-Sirius C19q-sgRNA targeting. Positive cell population is > 90% in CRISPR-Sirius C19q-sgRNA cells. No significant differences in growth rates were observed. NECTIN2 is located at 44.8 Mb and targeted 8 times by C19q-sgRNA. TOMM40 is located at 44.9 Mb and targeted 1 time by C19q-sgRNA. TNNT1 is located at 55.1 Mb and targeted 16 times by C19q-sgRNA. The gene locations are indicated in the diagram on the left side. C19q targeting region is marked by the red box

Previous studies showed that transcription activation can extend local chromatin fiber, as seen in the *HSP70* gene on bacterial artificial chromosomes in Chinese hamster ovary (CHO) cells, which is rescued by the transcription inhibitor, 5,6-dichloro-β-d-ribofuranosyl-benzimidazole (DRB) [32]. To investigate the impact of transcription on mesoscale chromosome conformation, we used DRB to inhibit transcription by blocking RNA Pol II elongation via CTD kinase inhibition [61]. Transcription inhibition was confirmed by measuring newly synthesized RNA with 5-ethynyl uridine (EU) in osteoblasts and osteosarcoma (OS) cells, with inhibition achieved within 2 h (Additional file 1: Fig. S2). We then recorded changes in chromosome conformation for 3 h during DRB treatment in both osteoblasts and OS cells (Fig. 2A and B). Surprisingly, transcription inhibition had no detectable effects on C19q conformational states in either cell type (Fig. 2 C, D and E, total number of cells, *n* $\ge$ 50, *p* > 0.05), suggesting that transcription does not regulate mesoscale chromatin organization. Similar results were reported in HT-1080 cells during early G1 stage [62]. Transcription inhibition by Actinomycin D did not affect the volume, surface area, and sphericity of human chromosome 11 territories. To examine whether targeting by CRISPR/dCas9 affects mRNA transcription, we measured the transcript levels of three active genes, *NECTIN2* (42.9 kb), *TOMM40* (12.4 kb), and *TNNT1* (16.5 kb), with and without CRISPR-Sirius targeting. These genes were targeted by C19q-sgRNA 8, 1, and 16 times, respectively. RT-PCR analysis confirmed that the targeting had no effect on transcript levels in OS cells (Fig. 2F).

### Osteosarcoma cells exhibit high levels of CTCF, cohesin and the euchromatin mark H3K27ac

**Fig. 3:**
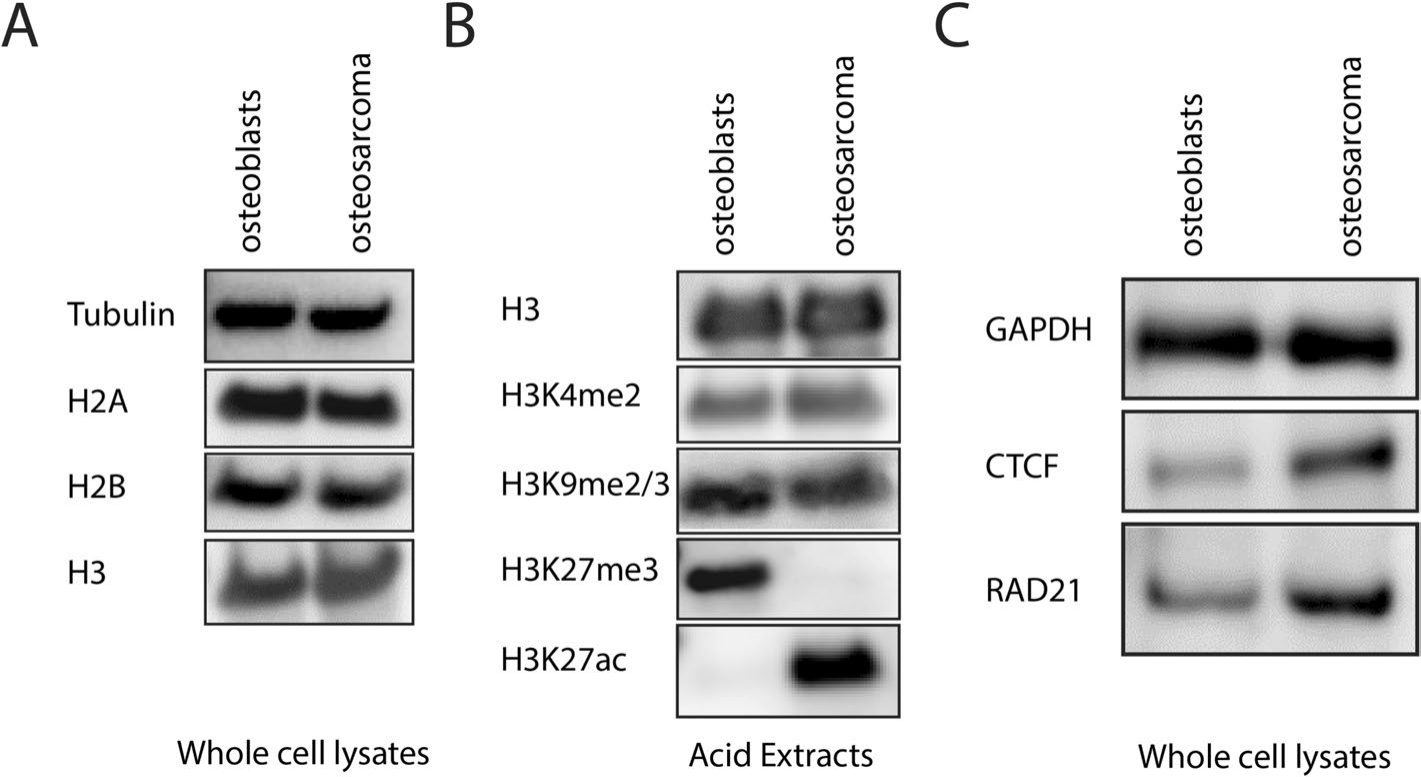
Different levels of chromatin architectural proteins and histone modifications were detected in osteoblast and osteosarcoma cells. **A** Western blotting of core histones. **B** Western blotting on the extraction of histone by acid extraction. While H3K9me2/3 and H3K4me2 levels are similar, a gain of H3K27ac and a loss of H3K27me3 were found in osteosarcoma. One million cells were used in each sample. **C** Western blotting shows that protein levels of CTCF and RAD21 (a cohesin subunit) are higher in osteosarcoma cells. All experiments were repeated at least three times

To investigate factors regulating mesoscale chromosome conformation in osteoblasts and OS cells, we examined core histones and histone PTMs, and chromatin architectural proteins, which control chromatin compaction, and mediate interphase chromatin looping, respectively. Histone PTMs, especially euchromatin and heterochromatin marks, can affect chromatin folding and dynamics [63]. Western blotting of whole-cell lysates and acid extracts revealed no significant differences in core histones H2A, H2B, and H3 between osteoblasts and OS cells (Fig. 3A). We further examined histone PTMs, focusing on the euchromatin marks H3K4me2/3 and H3K27ac, and heterochromatin marks H3K9me2/3 and H3K27me3. While H3K4me2/3 and H3K9me2/3 levels were similar, OS cells exhibited reduced H3K27me3 and increased H3K27ac compared to osteoblasts (Fig. 3B). H3K27me3, a facultative heterochromatin mark regulated by PRC2 complexes, is often misregulated in cancer and developmental disorders [64–67]. We hypothesized that maintenance of high H3K27me3 equilibrium levels is required to maintain the chromosome conformation in osteoblasts, which is absent in OS cells.

Western blot analysis of CTCF and RAD21 (the kleisin subunit of cohesin) revealed higher expression of both proteins in OS cells compared to osteoblasts (Fig. 3C). Given that RAD21 depletion impairs cohesin function and disrupts chromatin looping [68], we hypothesize that elevated levels of CTCF and cohesin in OS cells promote the collapsed chromosome conformations, with increased globularity, through extensive chromatin looping. In contrast, osteoblasts, which exhibit extended chromatin conformations, likely do not rely on CTCF/cohesin machinery to the same extent.

### Depletion of RAD21 alters gene expression and promotes extended chromosome conformation in osteosarcoma cells

**Fig. 4:**
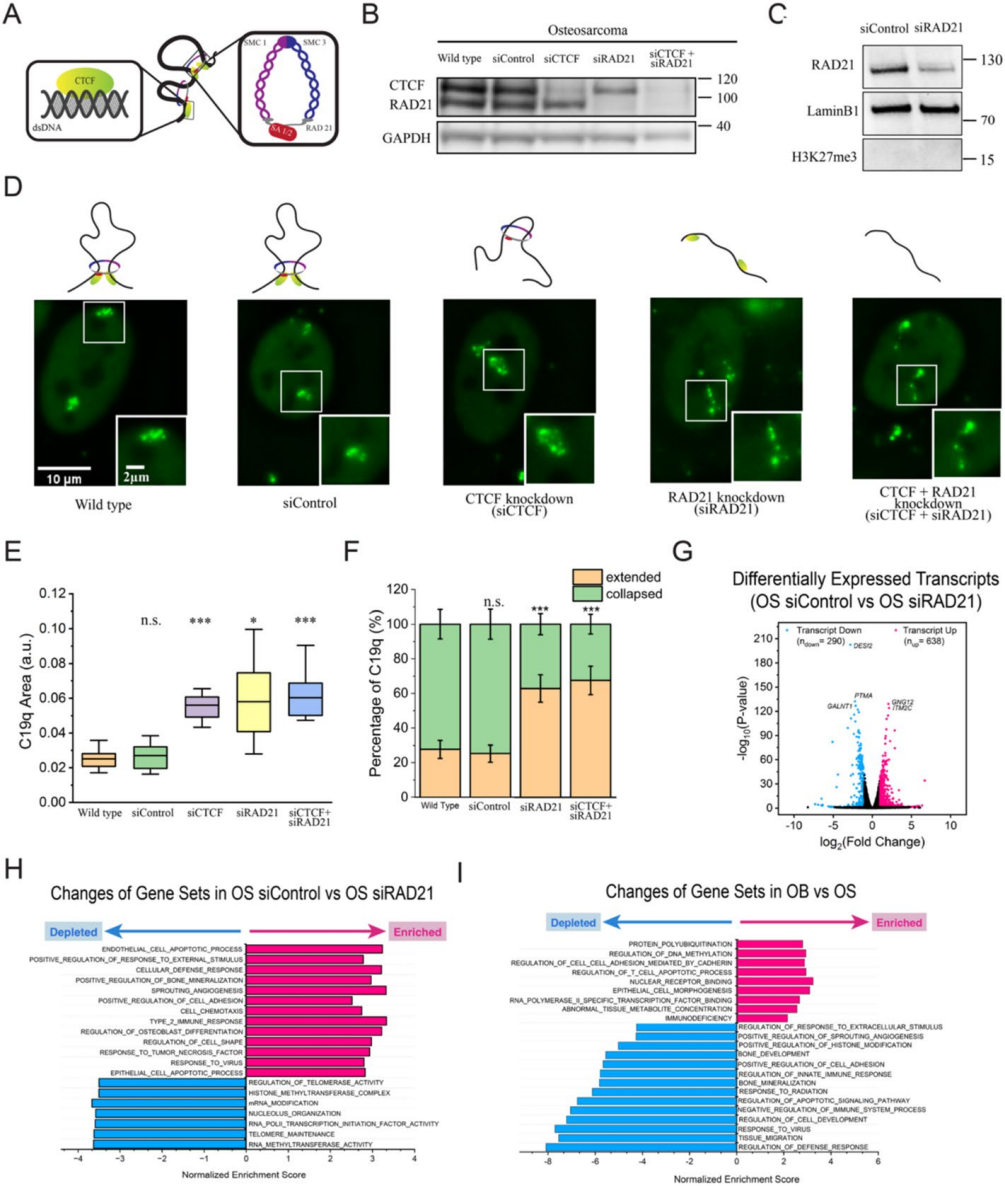
Increased chromatin looping correlates with collapsed C19q conformation in osteosarcoma and different gene profiles. **A** A schematic of chromatin architectural proteins, CCCTC binding factor (CTCF, green) and cohesin. RAD21 (grey) is the kleisin subunit of cohesin critical for ring formation. **B** Western blotting shows that CTCF and RAD21 protein expression levels were significantly reduced 24 hours post siRNA transfection in osteosarcoma cells (siCTCF and siRAD21, respectively). **C** Western blotting shows RAD21 knockdown does not change H3K27me3 levels in OS cells. **D** (left to right) Images of C19q in osteosarcoma cells in the indicated conditions. Images were taken 24 hours post-transfection for knockdown cells. **E** The area calculation of C19q in osteosarcoma cells upon siRNA transfection and negative control (siControl, N_C19q_=10 for each condition) **F** Percentages of cells with collapsed (green) and extended (orange) C19q conformations in osteosarcoma cells (N_C19q_=110 for wild type, N_C19q_=110 for negative control, N_C19q_= 110 for CTCF knockdown, N_C19q_= 110 for siRAD21, and N_C19q_= 110 for double knockdown) **G** Volcano plots of osteosarcoma cells with and without RAD21 knockdown (24 hours post-knockdown). Downregulated transcripts (log2FoldChange ⪳ −1, p<0.05) are colored in blue and upregulated transcripts (log2FoldChange ≧ 1, *p*<0.05) are colored in pink. Top 5 genes are labeled. **H** Genome wide gene set enrichment analysis (GSEA) of RNA seq with (siRAD21) and without (siControl) RAD21 knockdown (24 hours post-knockdown, ontology, *p*<0.05). **I** Genome wide gene set enrichment analysis (GSEA) of RNA seq in osteoblasts and osteosarcoma cells (ontology, *p*<0.05) . The significance tests in the Fig. 4E were calculated by using Welch’s T-test. The significance test in Fig. 4F was calculated by using Fisher’s exact test. *, *p*<0.05; **p<0.01, ***p<0.001; n.s., non-significant

To investigate the role of CTCF and cohesin in regulating chromosome conformations in OS cells, we used RNAi to knock down CTCF, RAD21, or both. CTCF and cohesin work concomitantly to establish and stabilize chromatin loops (Fig. 4A) [69, 70]. Western blotting confirmed successful knockdown of CTCF and RAD21 (Fig. 4B). Although enhanced regulation by the polycomb repressive system in cohesin-deficient mouse embryonic stem cells was previously reported [71], we observed that H3K27me3 levels remained low in RAD21-depleted OS cells, suggesting that H3K27me3 did not spread and does not play a major role in organizing C19q conformation in this context (Fig. 4C).

CTCF knockdown led to an expansion of the C19q area rather than an extension of its conformation (Fig. 4D). We observed a twofold increase in the C19q area in CTCF-knockdown OS cells, suggesting deregulated chromatin looping in the absence of the anchors (Fig. 4E). RAD21 knockdown induced extension of C19q conformations, further supporting the idea that excessive chromatin looping contributes to the collapse of C19q in OS cells (Fig. 4D, F, and Additional file 1: Fig. S3). A double knockdown of both CTCF and RAD21 also resulted in extended C19q conformations in OS cells, similar to RAD21 single knockdown (Fig. 4D and F). Cell viability was unaffected by single knockdowns of CTCF or RAD21 after 24 h, but the double knockdown reduced OS cell survival, indicating a potential dependency on both proteins for OS cell viability (Additional file 1: Fig. S4).

Next, we examined whether changes in chromosome conformation following RAD21 knockdown (siRAD21) were correlated with altered gene expression in OS. We performed RNA-seq analysis of OS cells with and without RAD21 knockdown at 24 h. Approximately 638 genes were upregulated, and 290 genes were downregulated genome-wide (absolute values of log2FoldChange ≥ 1, *p* < 0.05) (Fig. 4H). GSEA in oncology revealed that cancer related genes were abnormally expressed (Fig. 4I). Comparing GSEA data for siRAD21 cells with that for OS vs. osteoblasts (OB) showed opposing trends in genes related to bone mineralization, immune response, cell adhesion, and angiogenesis, suggesting a partial reversion of OS toward the OB phenotype. Notably, genes involved in the regulation of telomerase activity, such as *DKC1* and *MYC*, were downregulated, suggesting that RAD21 knockdown potentially reduces the lifespan of OS through telomere instability (Additional file 1: Fig. S5). These findings suggested that changes in chromosome conformation in RAD21-depleted OS cells are correlated with altered oncogenic gene expression, consistent with previous mechanistic and clinical genomic studies on the mutations of cohesion complexes or “cohesinopathies” as tumor drivers [72].

### Osteoblasts exhibit reduced chromatin mobility

**Fig. 5:**
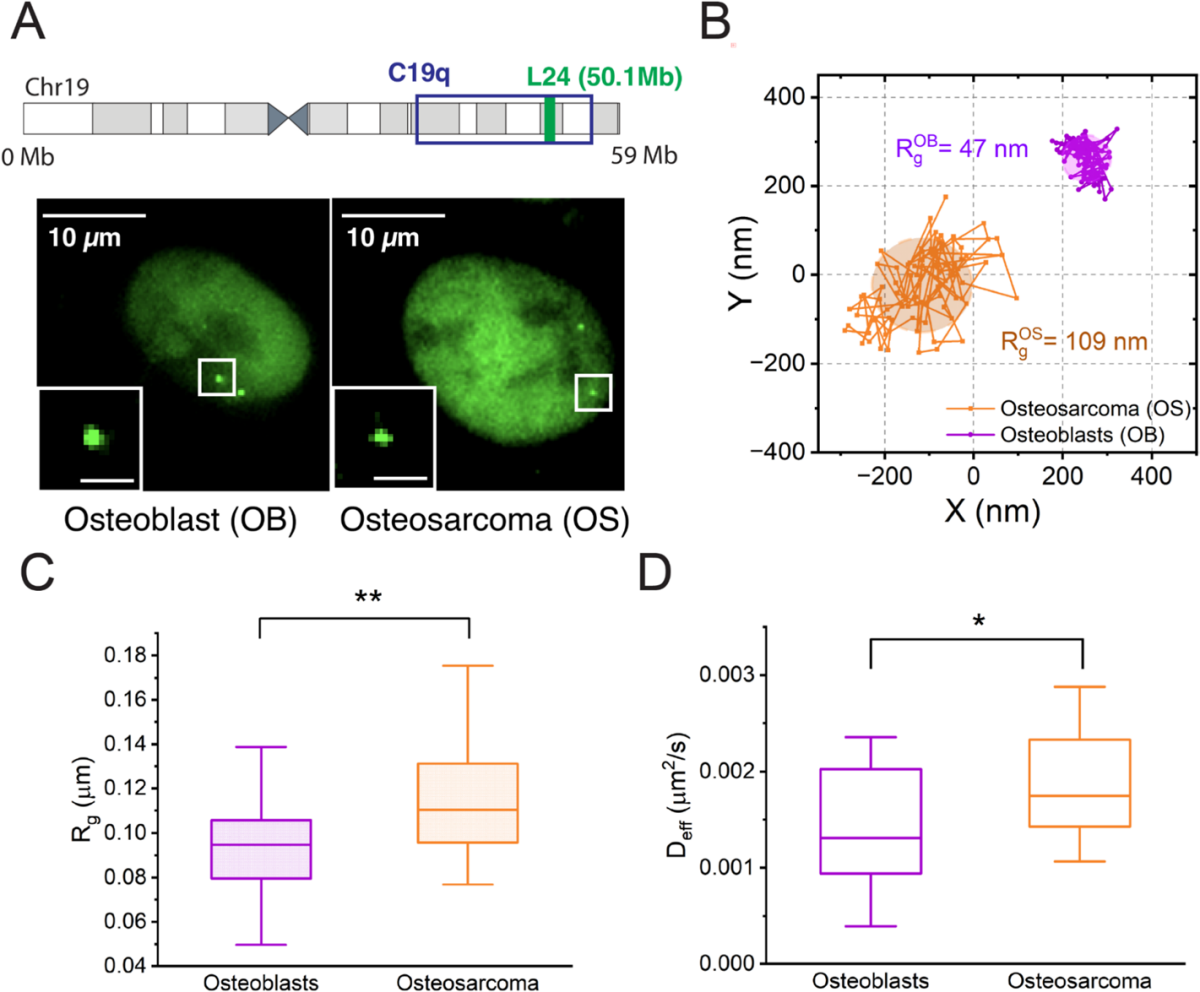
The H3K27me3-rich chromosome 19 in osteoblast cells is relatively less mobile. **A** Images of L24 loci in living cells by CRISPR-Sirius. Top: Location of L24 locus relative to the C19q targeting sites on the G-banding ideogram of Human chr 19; Left: osteoblasts; Right: osteosarcoma. Images were deconvoluted (2D) to reduce the background noise. Bar in the inset, 1 μm. **B** Typical L24 trajectories in osteosarcoma and osteoblast cells. Each trajectory was obtained by tracking the locus in a video containing 100 frames with 100 ms exposure time and 0.2 sec interval. **C** Box-and-whisker plot showing the gyration radii of L24 locus trajectories (R_g_) in the osteoblasts (*n*=24 trajectories, N_cell_= 21) and osteosarcoma (*n*=28 trajectories, N_cell_=23) cells. **D** Box-and- whisker plot showing the effective diffusion constants (D_eff_) of L24 loci in the osteosarcoma (*n*=28 trajectories, N_cell_=23) and osteoblast (*n*=24 trajectories, N_cell_=21) cells. Statistical significance was assessed by unpaired Welch's t-test with 95% confidence; *p<0.05 and **p<0.01. All experiments were repeated at least three times

Chromatin mobility reflects local chromatin material properties [73]. For example, nucleolus-associated heterochromatin is less mobile compared to other chromatin regions in HeLa cells, as observed by fluorescence correlation spectroscopy (FCS) [74]. Increased heterochromatin levels correlate with greater nuclear stiffness [75]. To investigate local chromatin material properties in osteoblasts and OS cells, we measured the mobility of the genomic locus L24, a locus with 15 copies of repeats located within C19q at 50.1 Mb on the linear genome. Using CRISPR-Sirius, we tracked L24 movement over time in both cell types (Fig. 5A, B). To quantify locus dynamics, we measured key biophysical parameters, including mean-square displacements (MSDs), trajectory radii (R_g_), and effective diffusion constants (D_eff_) (Fig. 5C, D and Additional file 1: Fig. S6). MSD and effective diffusion constants represent the locus mobility, while R_g_ (trajectory radii) reflects the radii of the areas traveled by locus within a given time. We found that the L24 locus in OS is significantly more mobile and has a larger R_g_ than those measured in osteoblasts. Additionally, the MSD curves in both cell types show a power-law dependence on lag time (Fig. 5C, D, and Additional file 1: Fig. S6). Previous studies investigating chromatin material properties via power-law MSD analysis using passive rheology have suggested that decreased chromatin mobility may correspond to an increased elastic modulus, suggesting a more solid-like chromatin state [76]. Based on this, we propose that chromosome 19 exhibits more solid-like behavior in osteoblasts compared to OS cells. However, additional studies are needed to more accurately characterize the chromatin material properties in both osteoblasts and OS cells.

### H3K27me3 is essential for maintaining the extended C19q conformation in osteoblast cells

**Fig. 6:**
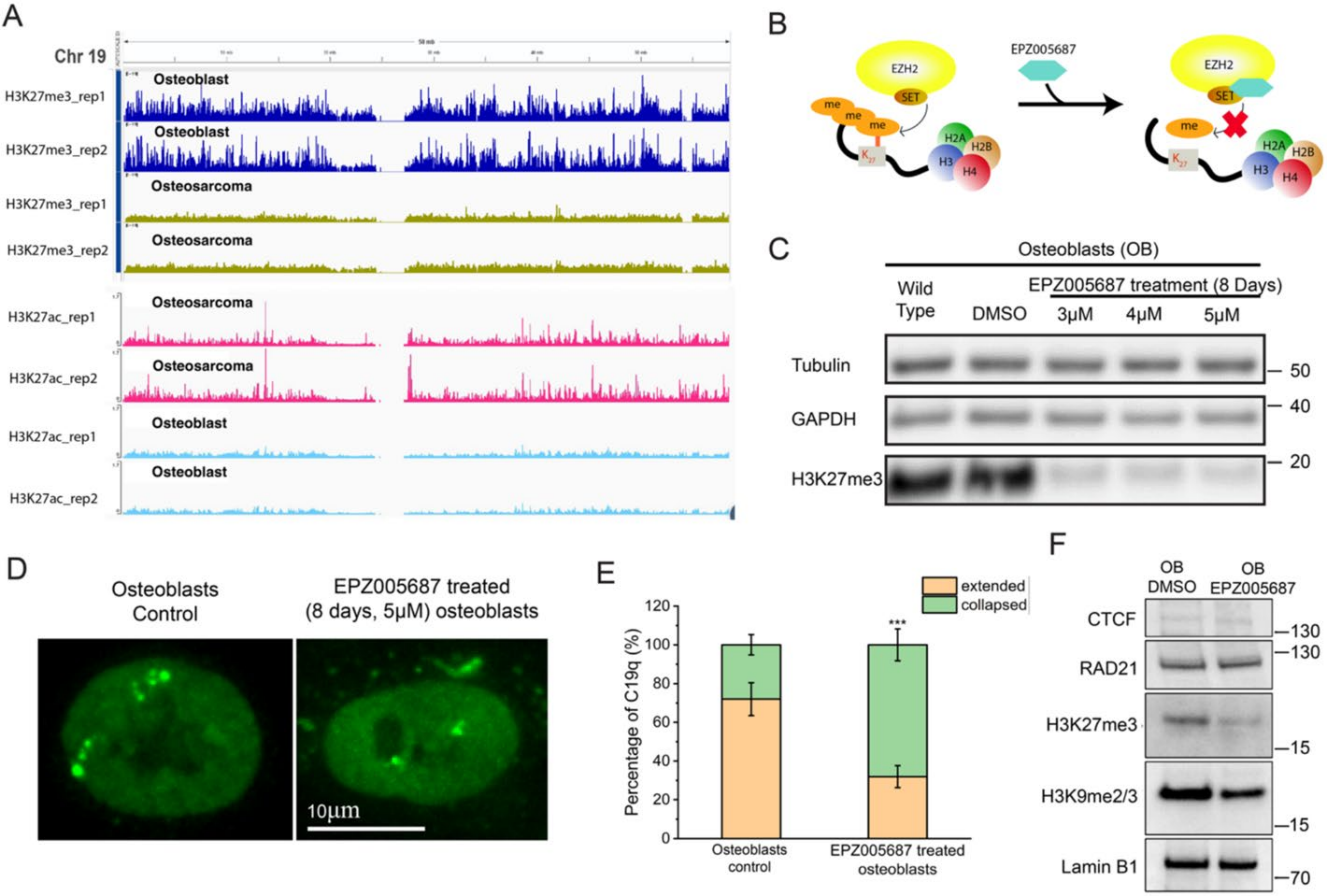
H3K27me3 is essential for maintaining extended C19q conformations in osteoblasts. **A** ChIP seq data showing H3K27me3 and H3K27ac occupancy along chr 19 in OB and OS cells. **B** Schematic diagram illustrating the action of EPZ005687, which prevents deposition of H3K27me3 by blocking the SET domain of EZH2 in PRC2 complexes. **C** Western blotting shows that H3K27me3 expression levels were significantly reduced within 8 days post EPZ005687 treatment in osteoblast cells. **D** Images of DMSO treated osteoblast cells (Osteoblasts Control) with extended C19q conformations and EPZ005687 treated osteoblast cells with collapsed C19q conformations. **E** Percentages of cells with collapsed (green) and extended (orange) C19q conformations in osteoblast cells under indicated conditions (N_C19q_=50 for each condition). The significance was calculated using Fisher’s exact test. *p<0.05, **p<0.01, ***, *p*<0.001. **F** Western blotting shows that reduction of H3K27me3 in OB upon EPZ005687 treatment does not change the levels of Rad21 and CTCF. However, a mild reduction of H3K9me2/3 was observed, suggesting collapsed C19q was not due to the increase of CTCF, cohesin, or H3K9me2/3

To confirm higher H3K27me3 levels in osteoblasts relative to OS cells, we performed spike-in ChIP-seq in both. Our results confirmed that H3K27me3 is enriched within the C19q region in osteoblasts but absent in OS cells (Fig. 6A). We then investigate whether H3K27me3 is essential for the maintenance of extended chromosomal conformations in osteoblasts. We tested this hypothesis by detecting chromosome conformations in osteoblasts with reduced H3K27me3 levels. Using the EZH2 inhibitor EPZ005687, which blocks H3K27me3 deposition by binding the SET domain of EZH2, we reduced H3K27me3 levels in osteoblasts [65] (Fig. 6B). Western blotting confirmed a significant reduction of H3K27me3 on day 8 in EPZ005687-treated osteoblasts (Fig. 6C). In these cells, the number of collapsed C19q conformations increased significantly compared to DMSO-treated control cells, which predominantly displayed extended conformations (Fig. 6D and Additional file 1: Fig. S7). Approximately 68% of EPZ005687-treated cells had collapsed C19q compared to 28% in untreated cells (Fig. 6E, *n* = 50, ***p < 0.001). As a control, we observed a reduction of H3K9me3 but no significant changes in RAD21 and CTCF levels in OB cells with reduced H3K27me3 (Fig. 6F). These findings suggest that TADs and the heterochromatin mark H3K9me3 do not play major roles in C19q collapse under reduced H3K27me3 conditions. Overall, our results demonstrate that H3K27me3 is essential for maintaining extended C19q conformations in osteoblast cells.

## Discussion

Transcription has been proposed to influence 3D chromatin organization by locally decompacting chromatin, thereby increasing accessibility [32, 77–79]. For instance, chromatin decompaction occurs during activation of the heat shock gene, *HSP70*, and large, highly expressed genes like *Ttn* [32, 80]. However, single-nucleosome tracking studies have shown that transcriptional inhibition using DRB can alter local chromatin dynamics without significantly affecting nucleosome domain structures [81], suggesting that while transcriptional changes at the gene level may influence local chromatin dynamics, they do not substantially alter mesoscale chromatin architecture. Consistently, we observed that transcription inhibition using DRB did not significantly alter C19q conformation, supporting the idea that transcription alone does not determine mesoscale chromosome architecture. Similarly, actinomycin D treatment did not affect the morphology of chromosome 11 territory in early G1 HT-1080 cells [62].

We found that chromosome 19q conformations differ significantly between OB and OS cells. OB cells predominantly display extended C19q conformations, associated with high levels of H3K27me3 and low levels of structural maintenance of chromosomes (SMC) complexes like cohesin. In contrast, OS cells contain primarily collapsed C19q configurations and show elevated levels of H3K27ac, CTCF, and cohesin. These findings align with prior reports linking reduced H3K27me3 in OS to elevated EZHIP, an inhibitor of PRC2 [82]. Although EZHIP knockdown alters gene expression, loss of H3K27me3 at promoters does not always increase H3K27ac or transcription [83], indicating that loss of repression may be insufficient for gene activation.

**Fig. 7:**
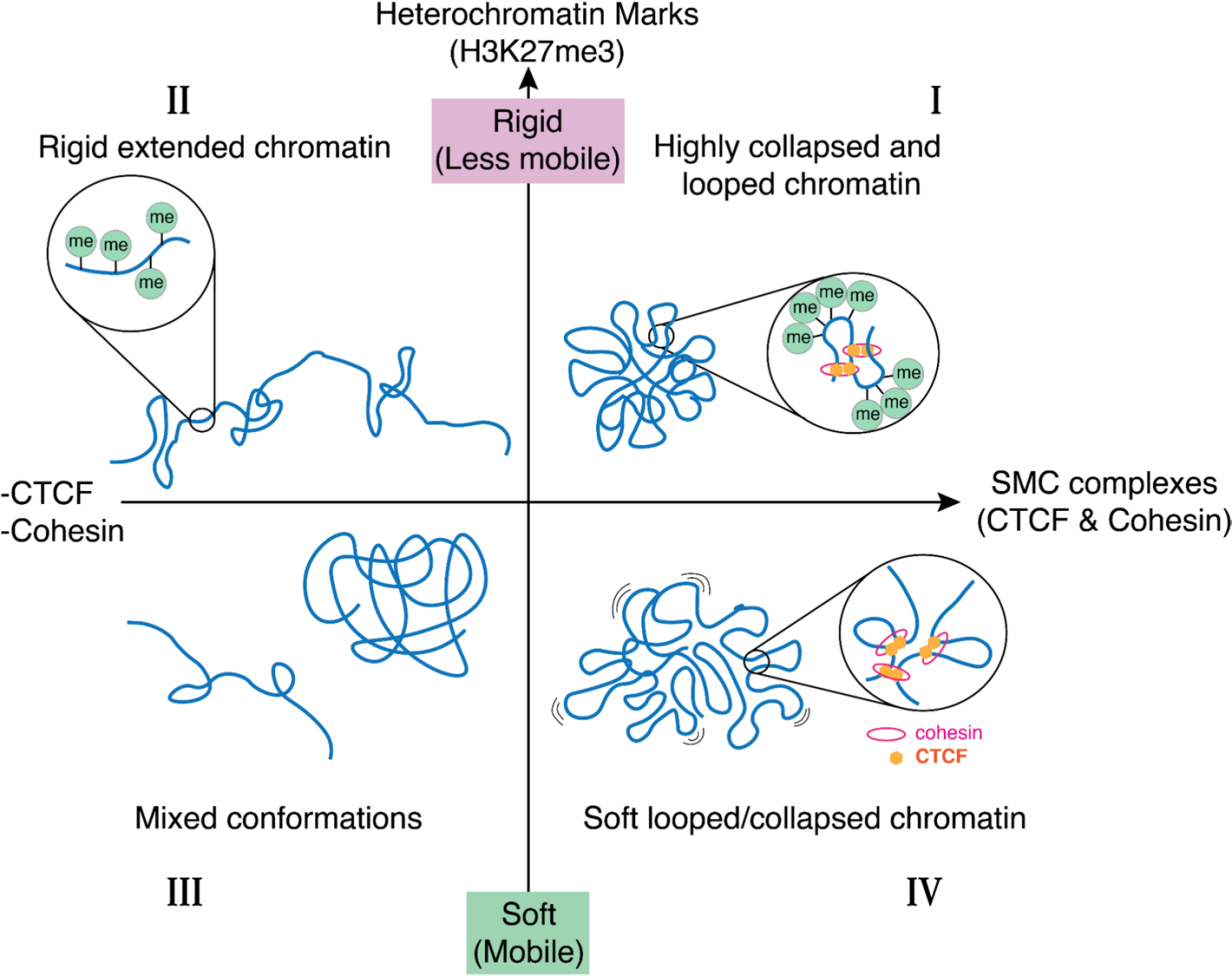
A model categorizing chromosome conformations in different conditions. Large-scale chromosome conformations are regulated by several factors. High levels of heterochromatin marks increase the rigidity of the chromatin, which results in stable conformations and slow mobility. On the other hand, the structural maintenance of chromosomes (SMC) complexes regulate the volume of mesoscale chromosomal domains by chromatin looping. In type I conformation, where both heterochromatin marks and SMC complexes are abundant, highly looped and rigid chromosomes result in highly collapsed conformations with minimal volume and mobility. The type II conformation is found in the nuclear environment with fewer SMC complexes but high levels of heterochromatin marks, making chromatin less bendable and more rigid. When both heterochromatin marks and SMC complexes are low, reduced constraints on chromatin lead to relatively unstable chromosome conformations that might switch between extended and collapsed conformations. The metastable conformations are likely determined by other factors, such as intrachromosomal interactions, chromatin-organelle attachments, or transcription activities. Finally, restricted by SMC complexes but not the heterochromatin marks, individual chromosomes form a collapsed but mobile conformation found in osteosarcoma cells (Type IV)

Importantly, recent super-resolution imaging and chromatin accessibility studies have challenged the idea that H3K27me3-rich chromatin only leads to more compact chromatin structure. For instance, some H3K27me3 marked regions may be similar or even less compact than unmarked regions [47]. Thus, the compaction observed upon EZH2 inhibition in our study may result from altered intra-chromatin interactions at the mesoscale, leading to conformational changes, rather than a simple local decompaction at smaller genomic scales. Our proposed model (Fig. 7) categorizes chromosome conformations based on the balance between heterochromatin marks and SMC complexes, offering a framework to understand chromatin territory architecture in different cellular contexts.

Our data demonstrate that CTCF knockdown leads to expansion rather than extension of the C19q conformation, and knockdown of RAD21 leads to an elongated C19q conformation in the OS cells. These results suggest that loop extrusion normally compacts chromatin locally and constrains spatial territory [84]. While TAD boundaries can influence gene regulation, their deletion in somatic cells often produces mild effects, indicating the presence of additional structural regulators [14, 33, 85]. Furthermore, although compartments are largely preserved in CTCF-depleted cells [86], the impact on the volume of individual chromosome territories is less clear. In G1E-ER4 cells, depletion of CTCF not only disrupted chromatin loops, but also altered compartmentalization in G1 by enhancing B–B inter-compartmental interactions and reducing A–A inter-compartmental interactions, resulting in increased loop size within A compartments, as determined by Hi-C and ChIP-seq [87]. Since C19q in OS is highly euchromatic and marked by H3K27ac, we speculate that the reduction in A–A inter-compartmental interactions and the increase in loop size contribute to the observed increase in chromatin volume on C19q under CTCF-depleted conditions. In contrast, such an increase in volume may not occur in heterochromatic regions, where enhanced interactions between B compartments are more likely to result in chromatin compaction. These findings underscore that large-scale chromatin organization is shaped by complex interplay among architectural proteins, histone modifications, and nuclear environment.

At the mesoscale, active loop extrusion, driven by cohesin, has been proposed to compete with compartmentalization through polymer brush effects [88]. Upon cohesin depletion, this balance shifts, potentially increasing spatial separation of A/B compartments. However, the implications for overall chromosome shape and territory size remain underexplored. In our study, cohesin depletion led to more elongated C19q structures, consistent with loss of loop-constrained compaction. Supporting this, live-cell imaging has shown that RAD21 depletion resulted in decompaction of nucleosome clusters [81], and increasing histone acetylation can decompact chromatin even in the presence of loop-extruding factors. This suggests that euchromatin marks may override loop extrusion in determining chromatin morphology.

We further observed that experimental conditions, such as temperature and humidity, may influence chromatin structure, underscoring the importance of maintaining optimal conditions during imaging and sample preparation. Specifically, we did not observe extended C19q in RAD21 knockdown cells when the samples were imaged at room temperature (~ 21 ℃) in the absence of CO_2_ and humidity supplies (Additional file 1: Fig. S8). Temperature has been critical for several cellular processes, including (1) chromatin dynamics, (2) RNA and protein folding, (3) transcription factor dimerization, and (4) protein phosphorylation [81, 89]. Incubation at a lower temperature may lead to the accumulation of misfolded RNA and proteins, including transcription activators and repressors, potentially altering chromatin conformations and transcriptional activity. Interestingly, significant changes in C19q conformation were not observed in the presence of chromatin looping factors such as cohesin, suggesting that TADs could facilitate genome stability under stressed conditions. Importantly, our CRISPR-based imaging method preserves endogenous chromosome organization by avoiding foreign DNA insertion and harsh chemical treatments.

Finally, we propose that C19q extension in osteoblasts is stabilized by H3K27me3, possibly by increasing structural rigidity, through two potential mechanisms: (1) tighter nucleosome packaging or local condensate formation, and (2) recruitment of chromatin reader proteins that restrict chromatin flexibility. Future studies on chromatin rigidity will help to clarify these mechanisms.

## Conclusions

Our study reveals distinct chromosome 19q (C19q) conformations and their regulations between osteoblasts (OB) and osteosarcoma (OS) cells, providing new insights into how chromatin architecture is regulated in disease. Using CRISPR-Sirius imaging, we show that OB cells exhibit extended C19q structures, while OS cells display more compact configurations. These differences correlate with altered gene expression in OS, including dysregulation of genes involved in immunity, bone biology, and metabolism—hallmarks of osteosarcoma.

Mechanistically, we found that the compact chromatin conformation in OS cells is associated with elevated CTCF and cohesin, suggesting enhanced loop formation. In contrast, OB cells maintain extended structures through high H3K27me3 levels, potentially by increasing chromatin rigidity. Transcription inhibition had minimal impact on C19q structure, indicating that transcription alone is not the primary determinant of mesoscale chromatin architecture. Notwithstanding the present findings, the immediacy of how transcription inhibition impacts chromatin organization and dynamics still needs further investigation, especially with regard to the different length scales that may be affected and their respective timescales.

Our findings underscore the complex and multifactorial nature of genome folding and its role in cancer. They also suggest that targeting chromatin regulatory proteins or histone modifications could offer novel therapeutic strategies for restoring normal chromatin architecture in osteosarcoma. Future research using patient samples and isogenic models will help determine whether similar alterations occur in other chromosomes or cancers and further define the role of 3D genome organization in disease progression.

## Methods

### Experimental details

#### Plasmid construction

The sgRNA (pLH-sgRNA-Sirius-8XPP7, Cat#121,940), dCas9 (pHAGE-TO-dCas9-P2A-HSA, Cat#121,936), and GFP-fused PP7 coat protein (pHAGE-EFS-PCP-GFPnls, Cat#121,938) expression constructs were obtained from Addgene. The design of these plasmids can be found in previous work [36, 37]. The sgRNA targeting sequences, TCCCTCAGACCCnGG for C19q and AGGTGGTTAGGAnGG for L24, were purchased from Integrated DNA Technologies, and cloned into the sgRNA vector using BbsI sites. The cloned sgRNA constructs were confirmed by Sanger sequencing.

#### Cell culture and transcription inhibition

Human bone osteosarcoma epithelial (U2OS), human embryonic kidney epithelial (HEK293T), and human osteoblast cell lines (hFOB 1.19) were acquired from ATCC. U2OS cells were maintained in DMEM (Corning) with high glucose supplemented with 10% FBS (Avantor) and 1% penicillin/streptomycin (Sigma) (Additional file 1: Table S1). HEK293T cells were maintained in Iscove’s modified Dulbecco’s medium supplemented with 1% GlutaMAX, 10% FBS, and 1% penicillin–streptomycin. The human osteoblast cell line was maintained in DMEM/F-12, 50:50 (Corning) media with l-glutamine supplemented with 10% FBS and 0.3 mg/ml G418. All the cell lines were confirmed to be free of mycoplasma contamination by using a Mycoalert PLUS kit (Lonza). To inhibit transcription, a final concentration of 50 µg/ml polymerase II elongation inhibitor 5,6-dichloro-1-β-ribofuranosylbenzimidazole (DRB; Sigma-Aldrich) was added to the culture medium and images were collected at different time points as indicated in Fig. 2.

#### Lentiviral transduction

To create stable cell lines with fluorescently labeled C19q, lentiviral particles carrying the sgRNA plasmids were generated using HEK293T cells as described previously [90]. Approximately 5 × 10^6^ cells were seeded in each well of a 6-well plate 24 h before transfection. Then, 0.5 µg of pCMV-dR8.2 dvpr (Addgene), 0.3 µg of pCMV-VSV-G (Addgene), and 1.5 µg of the sgRNA plasmid were cotransfected into the cell line using TransIT transfection reagent (Mirus) according to the manufacturer’s protocol [48]. Posttransfection, the virus was collected and filtered through a 0.45 µm filter. The virus was then immediately used or snap frozen and stored at − 80 °C. The hFOB 1.19 and U2OS cells maintained as mentioned above were transduced by spinfection in 6-well plates. Approximately 2 × 10^5^ cells were combined with 1 ml of lentiviral supernatant and centrifuged at 1200 × g for 30 min.

#### Flow cytometry

To select dCas9 and PCP-GFP positive cells, hFOB cells were sorted by fluorescence-activated sorting. The FACSAria Fusion Flow Cytometer (BD) was equipped with 405-, 488-, 561-, and 637-nm excitation lasers. PCP-GFP was detected with a 450/50 nm emission filter, and dCas9 positive cells were detected using an Alexa647-conjugated antibody to mouse CD24 (BioLegend) following the manufacturer’s staining protocol with a 670/30 nm emission filter.

#### EZH2 inhibitor treatment

Human osteoblast cells (hFOB1.19) were cultured at a confluence of 60–70% and then were split back to 30% confluence in fresh medium supplemented with the small molecule EZH2 inhibitor- EPZ005687 (selleckchem.com, CAT#: S7004) on days 3 and 6. The cells were incubated with EPZ005687 at 2 μM, 3 μM, 4 μM, and 5 μM in a total volume of 2 ml for days 7 and 8. Western blotting was used to confirm the protein expression levels of H3K27me3.

#### Fluorescence microscopy and image processing

A custom-built Olympus IX83 microscope equipped with three EMCCD cameras (Andor iXon 897), a LED, 60 × apochromatic oil objective lens (NA 1.5), and mounted with a 1.6 × magnification adaptor, resulting in a total Magnification of 96× . The microscope incubation chamber was Maintained at 37 °C supplied with 5% CO_2_ v/v and humidity for live cell imaging. Image data were acquired using CellSens software (version 4.1.1). For C19q imaging, 10 z-slices per nucleus were acquired with a step size of 0.25 µm and an exposure time of 100 ms. The images of a z-stack series were projected to a 2D image by using maximum intensity projection. Only cells that showed clear two or three well-separated C19q were included in the analysis. For each sample, approximately 50–75% of cells met this criterion and were analyzed in both cell lines. The image contrast was adjusted for optimal signal visibility.

The images were processed using Fiji [91] and Mathematica (Wolfram v12.3.1). To eliminate the drift movement of cells, the movement of individual genomic loci was calibrated by the motion relative to the nuclear centroid. The mean square displacement (MSD) of lag time kΔt was calculated by [92].

$$\text{MSD}(k\Delta t)=\frac{1}{(n-k+1)}\sum\limits_{m=0}^{n-k}{\left|p(m\Delta t+k\Delta t)-p(m\Delta t)\right|}^{2}$$

where p(t) is the position vector of a locus at time t, and Δt is a fixed time interval between two successive image frames. MSD curves were fitted using the power-law equation MSD(Δt) = 4Dapp Δt^α^, where D_app_ is the apparent diffusion constant. The gyration (or trajectory) radius R_g_ of the locus trajectory was calculated as:

$$R_g=\;\sqrt{\frac1{\left(n\;+\;1\right)}}\sum_{k=0}^n\vert P\left(t_k\right)\;-\;p_c\vert^2$$

where *p*_*C*_=${\sum }_{k=0}^{n}p({t}_{k})/(n+1)$ is the geometric center of the positions defining the trajectory and *t*_*k*+*1*_ = *t*_*k*_ + *Δt*. The gyration radius R_g_ measures the size of the area covered by locus movement. The effective diffusion constant D_eff_, is calculated by MSD at the short time intervals within 3Δt (MSD^(s)^) and fitted by the equation, MSD^(s)^ = 4D_eff_ Δt [39].

The C19q conformation or shape can be characterized by the aspect ratio of principal axes which is defined by the eigenvalues of gyration tensors. The components of gyration tensor $Q$ for N position vectors are defined by

$$Q_{\alpha \beta}=\;\frac1N\sum_{i=1}^N\left(r_{i,\alpha}-\;r_{c,\alpha}\right)\left(r_{i,\beta}-\;r_{c,\beta}\right)$$

where* r*_*i*_ and *r*_*c*_ denote individual denote individual position vectors and the position vector of the centroid of the N position vectors, $\alpha$ and $\beta$ represent the indices of the coordinate components of the position vector. For 2D data, $Q$ is a 2 × 2 symmetric matrix. We define the aspect ratio *K* of principal axes as $K={\lambda }_{1}/{\lambda }_{2}$ to characterize the conformation of C19q where ${\lambda }_{1}$ and ${\lambda }_{2}$ are the eigenvalues of $Q$ be and ${\lambda }_{1}\le {\lambda }_{2}$ [57, 58]. The larger *K*, the more extended the shape. The gyration tensor $Q$ of C19q was calculated from the spline curve of C19q output from Fiji [91]. Using the distribution of *K* values, we modeled C19q conformations by Gaussian bimodal fitting using OriginLab (version 2024b).

### Whole cell protein extraction

U2OS and hFOB cells were cultured in 6-well plates, trypsinized, and collected by centrifugation at 500 g for 3 min. The cell pellets were washed once with PBS. A total of 1 × 10^6^ cells were then resuspended in 250 µl of cell lysis buffer (1 × RIPA, 8 M urea and 1 × PMSF). The samples were then sonicated with a Diagenode Bioruptor (UCD-200) at medium intensity (200 W) for 5 min, cycling from 30 s ON to 30 s OFF. The protein concentration was determined using a Qubit protein kit (Invitrogen, Q-33211). The protein lysates were then aliquoted and stored at − 20 °C until further use.

### Acid extraction of histones

To extract histones, cells were cultured in 10 cm dishes, and 1 × 10^6^ cells were washed twice with ice-cold PBS and collected by centrifugation at 500 g for 3 min. The cell pellets were then resuspended in 1 ml of Triton Extraction Buffer (TEB, 0.5% v/v Triton X-100, 2 mM PMSF, 0.02% w/v NaN_3_ in PBS) followed by incubation on ice for 10 min. The cells were collected at 375 g at 4 °C for 10 min. Cell pellets were washed with 500 µl of TEB and collected again at 375 g for 10 min at 4 °C. The cells were then resuspended in 250 µl of freshly prepared 0.2 N HCl and incubated at 4 °C overnight for histone acid extraction. The samples were then centrifuged at 375 g and 4 °C for 10 min. The supernatant was transferred to a fresh 1.5 ml tube and the protein concentration was determined using a Qubit protein kit (Invitrogen, Q-33211). The protein lysates were stored at − 20 °C until further use.

### Antibodies and Western blots

Fifty micrograms of whole cell lysates and 5 µl of acid extracts were separated on precast Bis–Tris protein gels (Bolt Bis–Tris Plus gels) and transferred to PVDF membranes (Bio-rad). Rabbit polyclonal antibodies against CTCF (Cat#2899S), H2B-V119 (Cat#8135S), H3 (Cat#9715S), and rabbit monoclonal antibody against H2A-D603A (Cat#12349S) were obtained from Cell Signaling Technology. Rabbit polyclonal antibodies against RAD21 (Cat#27,071–1-AP), GAPDH (Cat#10,494–1-AP), and alpha-Tubulin (Cat#11,224–1-AP) were obtained from Proteintech. Rabbit polyclonal antibody for H3K9me2/3 (Cat#ab8898) was obtained from Abcam. Rabbit polyclonal antibody against H3K27me3 (Cat#39,155) was obtained from Active Motif.

### siRNA transfection

U2OS cells were seeded at 6 × 10^4^ cells per well in a 24-well glass-bottom plate or at 2 × 10^4^ cells in the microscope imaging dish. The siRNAs against CTCF (ID: s20966, cat:4,392,420), RAD21 (ID: s531212, cat:4,392,420), and negative control #1(cat:4,390,843) were obtained from Ambion. The cells were then transfected upon reaching 60% confluency using TransIT transfection reagents (Mirus). The images were taken 24 h post-transfection.

### RT-PCR

Total RNA was extracted by using a RNeasy plus mini kit (QIAGEN), then first-strand cDNA was synthesized by using the SuperScript IV reverse transcriptase (Invitrogen) according to the supplier’s instructions. β-ACTIN was used as an internal control for RT-PCR analysis. DNA was stained by ethidium bromide. DNA oligonucleotides used in this study: NECTIN2-F: TGCCCTCCCAGCTCTTCACT, NECTIN2-R: TTGTGGCTCCAGGGTGCAAG, TOMM40-F: GGACACCAGCGTCTCCTTCG, TOMM40-R: CGGTGATTCAGGAAGGCCCC, TNNT1-F: AAGCGGGCAGAGGATGATGC, TNNT1-R: AGCTGGTGGATCCAGTCCGA, β-ACTIN-F: GTGGGGCGCCCCAGGCACCA, and β-ACTIN-R: CTCCTTAATGTCACGCACGATTTC.

### RNA seq analysis

The raw data of RNA-seq for RAD21 knockdown (GEO accession no. GSE89799, SRA–SRX2344649, SRX2344650, SRX2344659, and SRX2344660) and hFob1.19 (GEO accession no. GSE118488, SRA–SRX4549277, SRX4549278) and untreated U2OS (GEO accession no. GSE118488, SRA–SRX4549306 and SRX4549307) were obtained from Sequence Research Archive [93]. The data analysis, including DESeq and GSEA, was performed on SciDAP (https://scidap.com/).

### Chromatin immunoprecipitation

The U2OS and hFOB cells were cultured in 10 cm dishes and trypsinized. About 3.15 × 10^6^ cells were collected by centrifugation at 500 g for 5 min and then washed with PBS. The cell pellets were resuspended in 10 ml fixing buffer (50 mM Hepes pH 8.0, 1 mM EDTA, 0.5 mM EGTA, 100 mM NaCl). 16% w/v methanol-free formaldehyde (Thermo Fisher Scientific, Cat#28,906) was added to a final concentration of 1% for cell fixation. After an incubation of 10 min at room temperature, the reaction was quenched using glycine at a final concentration of 125 mM. The cell suspension was placed on ice for 5 min followed by centrifugation at 1200 g at 4 °C for 5 min and the supernatant was discarded. The 2 × 10^6^ formaldehyde fixed mouse C2C12 myoblasts were spiked in with each sample containing 3.15 × 10^6^ human cells resuspended in NP1 Rinse 1 buffer (50 mM Hepes pH 8.0, 140 mM NaCl, 1 mM EDTA, 10% glycerol, 0.5% NP-40, 0.25% Triton X-100). Samples were incubated for 10 min at room temperature and pelleted at 1200 g for 5 min at 4 °C. The cell pellets were resuspended in NP Rinse 2 buffer (10 mM Tris–Cl pH 8.0, 1 mM EDTA, 0.5 mM EGTA, 200 mM NaCl) followed by centrifugation at 1200 g for 5 min at 4 °C. Finally, the cells were washed with Covaris Shearing buffer to remove any salts. The cells were then resuspended in 900 µl of Covaris shearing buffer after adding freshly prepared protease inhibitor cocktail (Sigma, Cat# 11,697,498,001) to the Covaris shearing buffer. The cells were then transferred to 1 ml Covaris shearing tube (Covaris milliTUBE 1 ml AFA Fiber, 520,135). The cells were sonicated using Covaris sonicator (E220 evolution) for 8 min at a cycle of 30 s ON and 30 s OFF. Then, 12.5 µl of the sonicated chromatin stock was added to 12.5 µl of Covaris shearing buffer along with 10 µg RNase A (Thermo Scientific, EN0531) and incubated at 37 °C for 30 min. Further, 10 µg of proteinase K (Zymo Research, D3001-2–20) was added to this input sample followed by an overnight incubation at 65 °C. The input sample was then purified using MinElute PCR purification columns (QIAGEN, 28,004). To check the shearing, 5 µl of eluted DNA was run on E-gel 2% EX agarose gel (Invitrogen, G401002) for 10 min. The remaining 960 µl sheared chromatin lysate was centrifuged at 10,000 g at 4 °C for 5 min. Combine the supernatant with 240 µl of 5 × IP buffer (250 mM HEPES/KOH pH 7.5, 1.5 M NaCl, 5 mM EDTA, 5% Triton X 100, 0.5% Sodium Deoxycholate, 0.5% SDS). Moreover, 5 µl of H3K27me3 antibody (Active motif, Cat#39,155) was added to each sample. For each sample, 40 µl of Protein G magnetic Dynabeads were buffer exchanged with ChIP buffer (50 mM HEPES/KOH pH 7.5, 300 mM NaCl, 1 mM EDTA, 1% Triton-X 100, 0.1% sodium deoxycholate, 0.1% SDS) added to the sample after the antibody was added. The antibody: chromatin mixture was incubated overnight at 4 °C with overhead rotation. The beads were then washed twice with ChIP buffer, once with DOC buffer (10 mM Tris–Cl pH 8.0, 0.25 M LiCl, 0.5% NP40, 0.5% sodium deoxycholate, 1 mM EDTA) and once with TE buffer pH 7.4. The beads were resuspended in 100 µl TE buffer pH 7.4 with 2.5 µl 10% SDS and 5 µl 10 mg/ml Proteinase K. The ChIP samples were then reverse crosslinked at 65 °C overnight. The supernatant was purified with QIAGEN MinElute PCR purification columns.

The ChIP was validated using real-time PCR with primers designed against HOXB7 (forward–5′-GAGTAACTTCCGGATCTACCC-3′, reverse–5′-CGTCAGGTAGCGATTGTAGTG-3′ known to be enriched with H3K27me3 and GAPDH (forward–5′-ACATCGCTCAGACACCATG-3′, reverse–5′-TGTAGTTGAGGTCAATGAAGGG-3′ as a negative control. The primers were purchased from IDT. For validation, 1 µl of the input sample or ChIP sample along with validation primers and SYBR green master mix (Biorad, 1,725,270) was added in the BioRad RT-PCR plates. The reaction mix was incubated in Biorad CFX96 Real time system with C1000 thermal cycler module at standard RT-PCR conditions [94]. Ct values from both the negative and positive control were used to calculate fold enrichment using the ΔΔCt method [94].

### ChIP library construction and next generation sequencing

ChIP and input DNA from both U2OS and hFOB 1.19 were prepared for sequencing as directed by Lucigen End-It DNA repair Kit, followed by 3′ A-tailing by Klenow exo (NEB, M0212L) and adaptor ligation using T4 DNA ligase (NEB, M0202L). The Linker Ligated DNA was size selected on an E-gel 2% EX agarose gel. The libraries were amplified using barcoded primers followed by gel purification to remove primer dimers or unreacted primers by gel purification. Pooled libraries were validated using Qubit and sequenced at the Nationwide Children’s Hospital Institute for Genomic Medicine (NCHIGM) on a HiSeq4000 sequencer to obtain 150 bp paired end reads.

### ChIP-seq data analysis

Based on the ratio of spike-in mouse cells and human cells, we performed read-depth correction by normalizing all input and ChIP samples using the mouse-spike-in reads. The raw fastq files obtained from NCHIGM were checked for quality using the FastQC v.0.72 [95]. The fastq reads were aligned separately to the mouse genome build mm10 (GRCm38) and the human genome build hg38 (GRCh38) using the bioinformatic tool Bowtie2 v.2.4.2 [96], to generate the BAM files. To calculate the scaling factor (f) for normalizing the samples, the minimum number of mice aligned reads (m) were identified across all samples. The scaling factor was calculated by dividing the minimum number of mice aligned reads by the number of aligned mice reads for each sample (s) – f = m/s. Samtools View[97] -s -b was used to subsample the hg38 aligned BAMs as it retains the read pair information. The subsampled BAM files were then converted to paired end fastq files using the Picard SamToFastq v.2.18.2 [98].

The normalized subsampled reads were aligned to hg38 using Bowtie2 v.2.4.2 [96] with parameters bowtie2 -X2000. BAM sort was used to sort the BAM files. The list of blacklist regions compiled by ENCODE consortia–hg38-blacklist.v2.bed.gz was used to remove blacklist regions[99]. Similarly, unmapped, mate unpaired, multi-mapped, duplicate reads, PCR duplicates, and low mapping quality reads (MAPQ > 30) were removed using ngsutils filter v.0.5.9 [100] and bamtools filter v.2.4.0 [101]. MACS2 was used to call peaks on the filtered data using the following parameters MACS2 -p 1e-2 -nomodel -shift 0 -extsize $[FRAGLEN] -keep-dup all -broad -SPMR, where FRAGLEN is the estimated fragment length based on your library size [102]. For visualization, bedGraph files were generated with MACS2 bdgcmp from the pile-up, and then converted to bigwig format using BedGraphtobiWig and visualized using Integrative Genomics Viewer (IGV) [103].

### Quantification and statistical analysis

All analytical and statistical tests other than ChIP-seq, RNA-seq, and chromatin mobility (Figs. 5 and S6) were performed using R (4.0.5) [104]. Statistical tests for Fig. 5 and Additional file 1: Fig. S6 were performed using OriginPro (version 2024b).

## Supplementary Information

Additional file 1: Table S1. A list of key resource, including reagents, software, and cell lines. Fig. S1. Distinct chromosome conformations in synchronized osteosarcoma cells. Fig. S2. DRB treatment significantly reduced newly synthesized RNA in osteoblasts and osteosarcoma cells. Fig. S3. Distributions of aspect ratio K in OS cells with and without siRNA knockdown of RAD21 and CTCF. Fig. S4. Cell viability in osteosarcoma upon RNAi treatment. Fig. S5. Heatmap of dysregulated genes associated with regulation of telomerase activity upon RAD21 knockdown in osteosarcoma cells. Fig. S6. Power-law MSD curve of locus dynamics. Fig. S7. Distributions of aspect ratio K in OB cells with and without EPZ005687 treatment. Fig. S8. C19q chromosome conformations are sensitive to cell survival conditions.

Additional file 2. Uncropped western blot images and RT-PCR gel image.

Additional file 3: Movie S1: A typical movement of the C19q in the osteoblast.

Additional file 4: Movie S2: A typical movement of the C19q in the osteosarcoma

## Data Availability

The Raw ChIP-seq sequence files prepared for this manuscript are publicly available through the Gene Expression Omnibus under accession number BioProject PRJNA1111443. The raw image data for this paper are publicly available through the BioImage Archive [105, 106] (http://www.ebi.ac.uk/bioimage-archive) under accession number S-BIAD2272. All publicly available data have been cited in the paper. Uncropped western blot images and RT-PCR gel image are provided as supplemental material (Additional file 2). Other data reported in this paper are available from the lead contact on reasonable request.
