# Supplementary material for: Differential regulation of mesoscale chromosome conformations in osteoblasts and osteosarcoma
Source: Genome Biol. 2025 Sep 26;26:307. doi: 10.1186/s13059-025-03785-2 (PMC12465978; doi:10.1186/s13059-025-03785-2)
Supplement: Supplementary file 1 — Additional file 1: Table S1. A list of key resource, including reagents, software, and cell lines. Fig. S1. Distinct chromosome conformations in synchronized osteosarcoma cells. Fig. S2. DRB treatment significantly reduced newly synthesized RNA in osteoblasts and osteosarcoma cells. Fig. S3. Distributions of aspect ratio K in OS cells with and without siRNA knockdown of RAD21 and CTCF. Fig. S4. Cell viability in osteosarcoma upon RNAi treatment. Fig. S5. Heatmap of dysregulated genes associated with regulation of telomerase activity upon RAD21 knockdown in osteosarcoma cells. Fig. S6. Power-law MSD curve of locus dynamics. Fig. S7. Distributions of aspect ratio K in OB cells with and without EPZ005687 treatment. Fig. S8. C19q chromosome conformations are sensitive to cell survival conditions. [file 13059_2025_3785_MOESM1_ESM.docx]

**Additional file 1**

**Differential regulation of mesoscale chromosome conformations in osteoblasts and osteosarcoma**

**Madhoolika Bisht^1,2,^**^†^**_,_ Yu-Chieh Chung^1,^**^†^**, Siou-Luan He^1^, Sydney Willey^1,7^, Benjamin D. Sunkel^5^, Meng Wang^5^, Benjamin Z. Stanton^1,5,6^, Li-Chun Tu^1,3,4,7,*^**

^1^Department of Biological Chemistry and Pharmacology, The Ohio State University, Columbus, OH, USA 43210

^2^Department of Molecular Genetics, The Ohio State University, Columbus, OH, USA 43210

^3^Center for RNA Biology, The Ohio State University, Columbus, OH, USA 43210

^4^The Ohio State University Comprehensive Cancer Center, The Ohio State University, Columbus, OH, USA 43210

^5^Nationwide Children’s Hospital, Center for Childhood Cancer and Blood Diseases, Columbus, OH 43205

^6^Department of Pediatrics, The Ohio State University College of Medicine, Columbus, OH, USA 43210

^7^7Present address: Michigan Neuroscience Institute, University of Michigan, Ann Arbor, MI 48109.

†These authors contributed equally to the work.

*Correspondence: Li-Chun Tu, **Tu.277@osu.edu**

**This Additional file 1 includes:**

**Figures S1 to S8**

**Legends for Movies S1 and S2**

**Table S1**

**Other supplementary materials for this manuscript include the following:**

**Movies S1 to S2**

**Additional file 2**

**
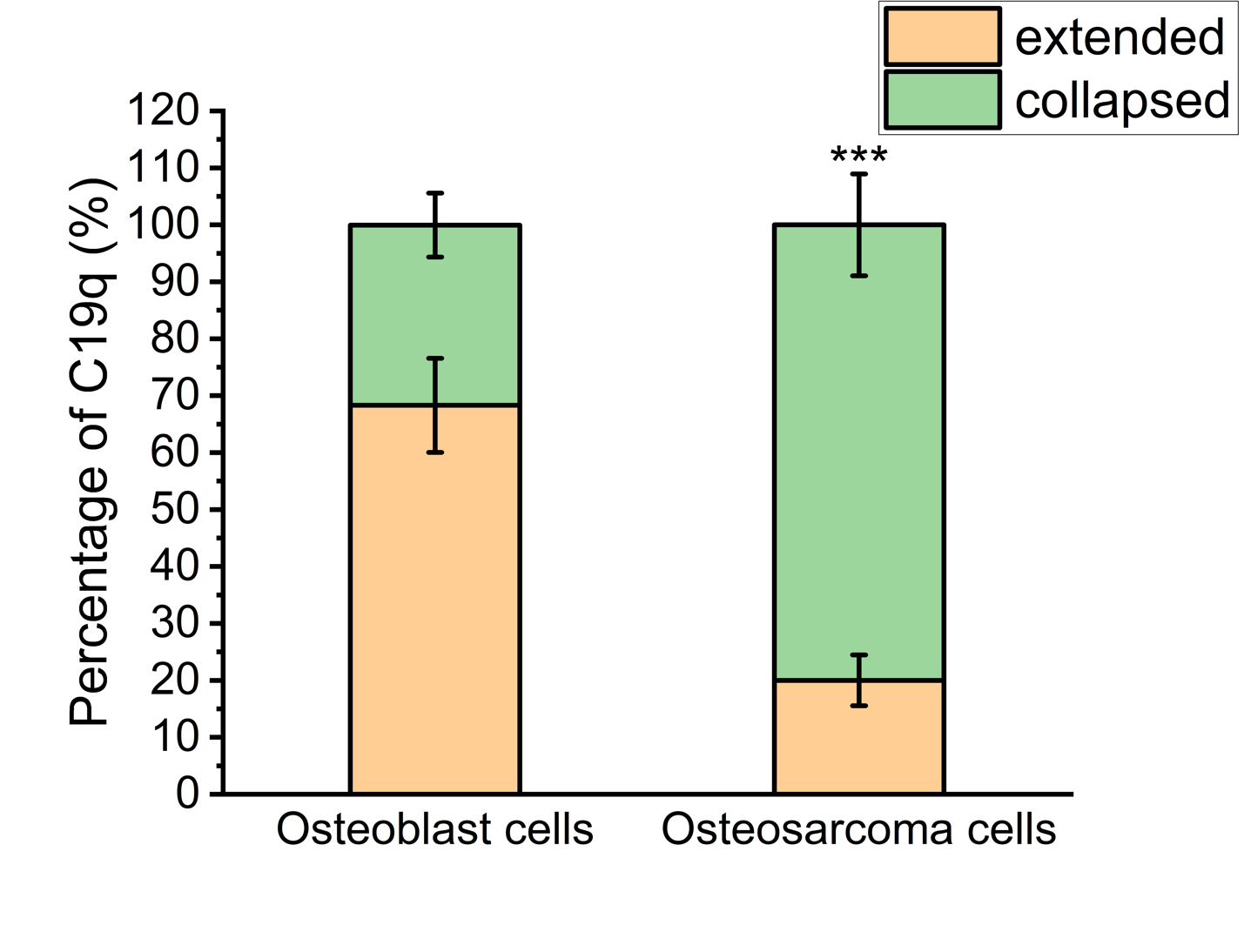
 Figure S1. Distinct chromosome conformations in synchronized osteosarcoma cells.** Percentages of cells with collapsed (green) and extended (orange) C19q conformations in osteosarcoma cells synchronized to mid-late G1 phase (N_C19q_=60 for each sample). The significance test was calculated using Fisher’s exact test. ***, p<0.001.

**
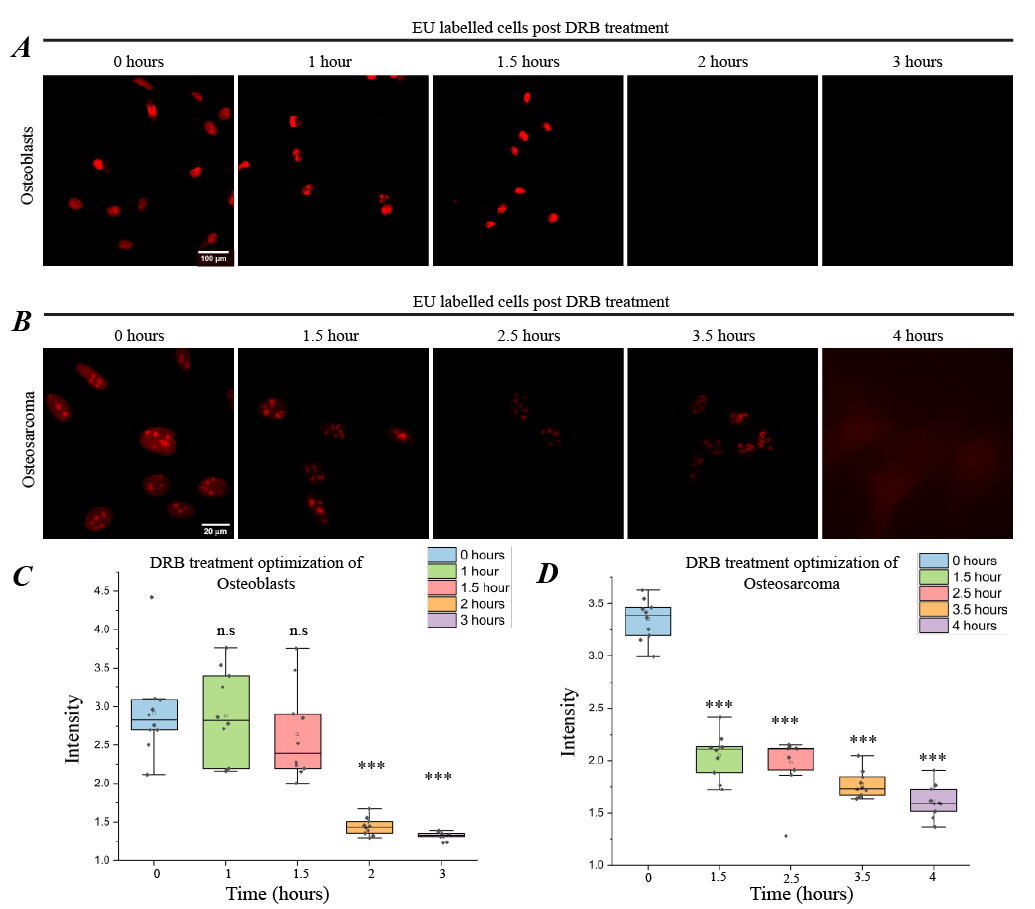
**

**Figure S2. DRB treatment significantly reduced newly synthesized RNA in osteoblasts and osteosarcoma cells.**

5-ethynyl uridine (EU) was used to detect newly synthesized RNA during transcription inhibition using DRB in (A) osteoblast cells and (B) osteosarcoma cells. Prolonged EU incubation induced extensive cell death. Intensity measurement of the EU signal in (C) osteoblasts and (D) osteosarcoma cells (N_C19q_ =10 for each condition). The significance was calculated using Welch’s T-test: significant difference, *, p<0.05; **, p<0.05, ***, p<0.001; N.S., non-significant.


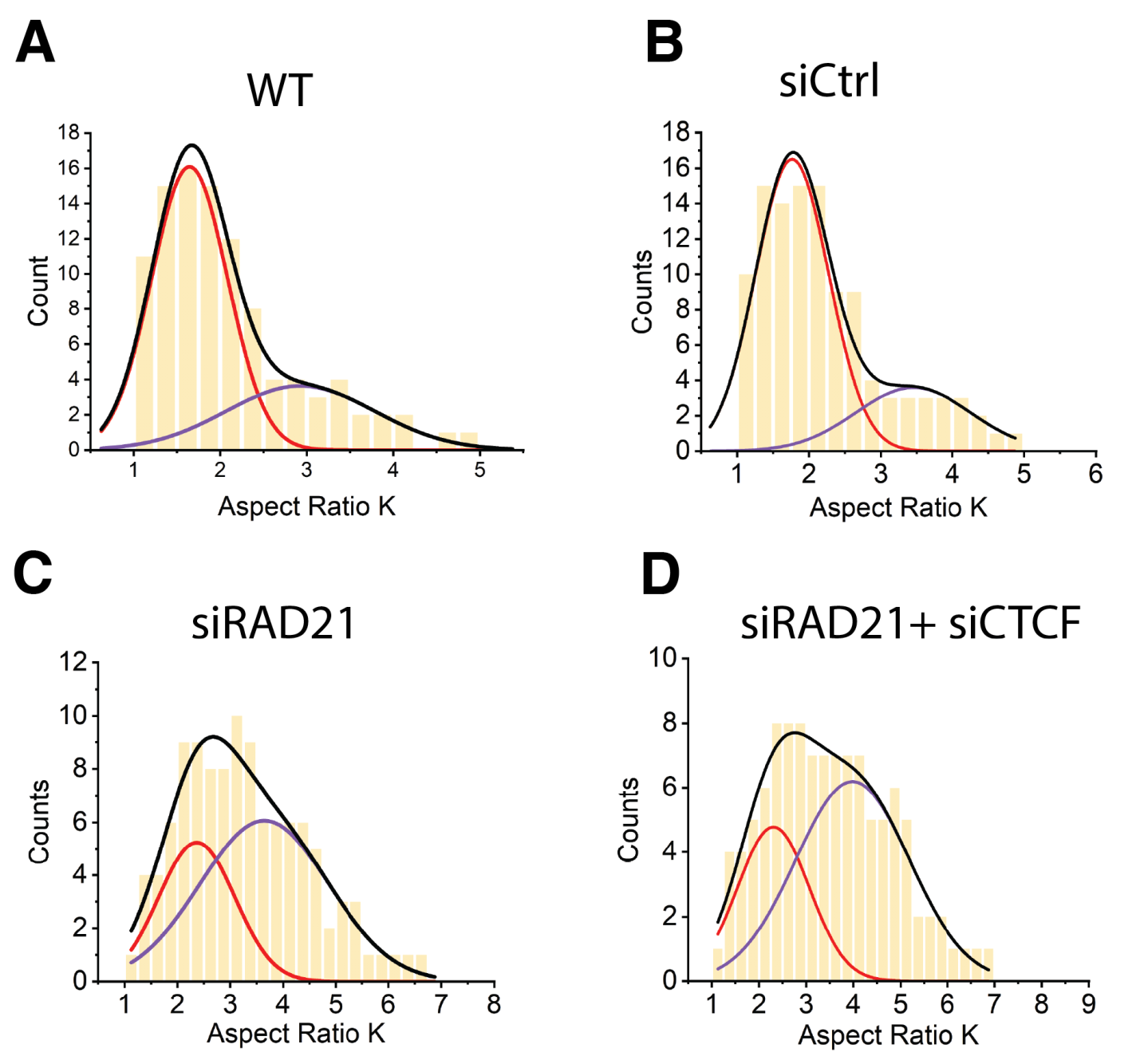


**Figure S3. Distributions of aspect ratio K in OS cells with and without siRNA knockdown of *RAD21* and *CTCF*.**

Gaussian bimodal fitting of aspect ratio (K) distribution of C19q conformations for osteosarcoma (OS) cells under different siRNA knockdown conditions: (A) Wild type (WT, N_C19q_= 100) (B) Negative control (siCtrl, N_C19q_=110) (C) RAD21 knockdown (siRAD21, N_C19q_= 110) (D) RAD21 and CTCF double knockdown (siRAD21+siCTCF, N_C19q_= 110). The black line represents the overall bimodal Gaussian fit. Purple and green lines represent the individual Gaussian distributions corresponding to collapsed and extended conformations, respectively.


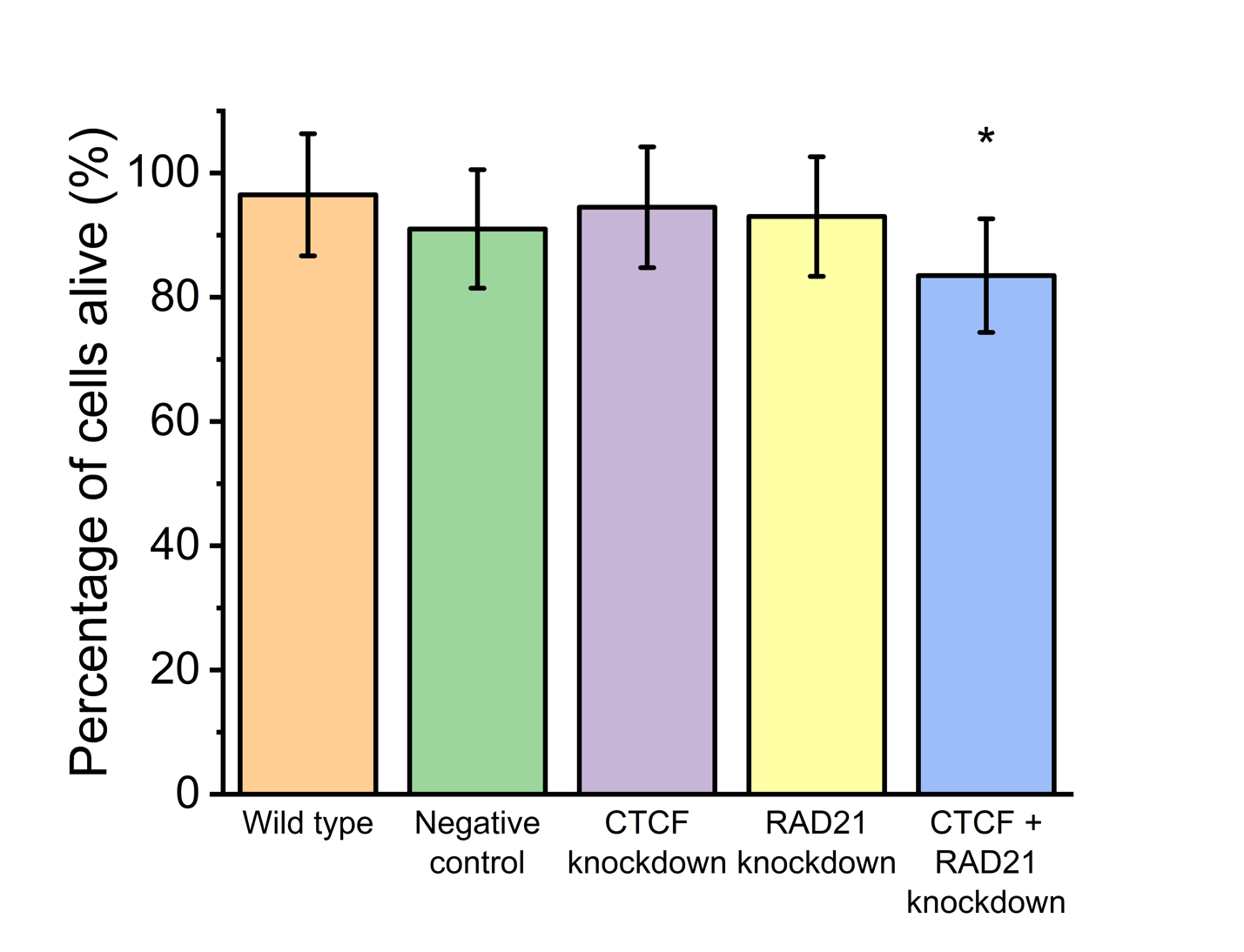


**Figure S4: Cell viability in osteosarcoma upon RNAi treatment.**

Plot shows that osteosarcoma cells have no significant change in viability upon RNAi with a negative control, CTCF and RAD21 knockdown for 24 hours post-transfection. However, the cell viability decreases in osteosarcoma cells upon double knockdown of both CTCF and RAD21(*, p-value < 0.05). P-values in the figure are calculated by using Chi-square test with Yates correction.


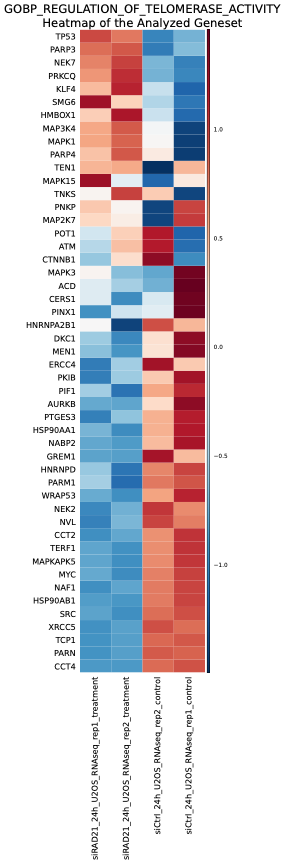


**Figure S5: Heatmap of dysregulated genes associated with regulation of telomerase activity upon RAD21 knockdown in osteosarcoma cells**

GSEA analysis in ontology showed that genes associated with regulation of telomerase activity were largely depleted upon RAD21 knockdown at 24 hours in osteosarcoma cells. Upregulated genes are colored in red and downregulated genes are colored in blue.

**
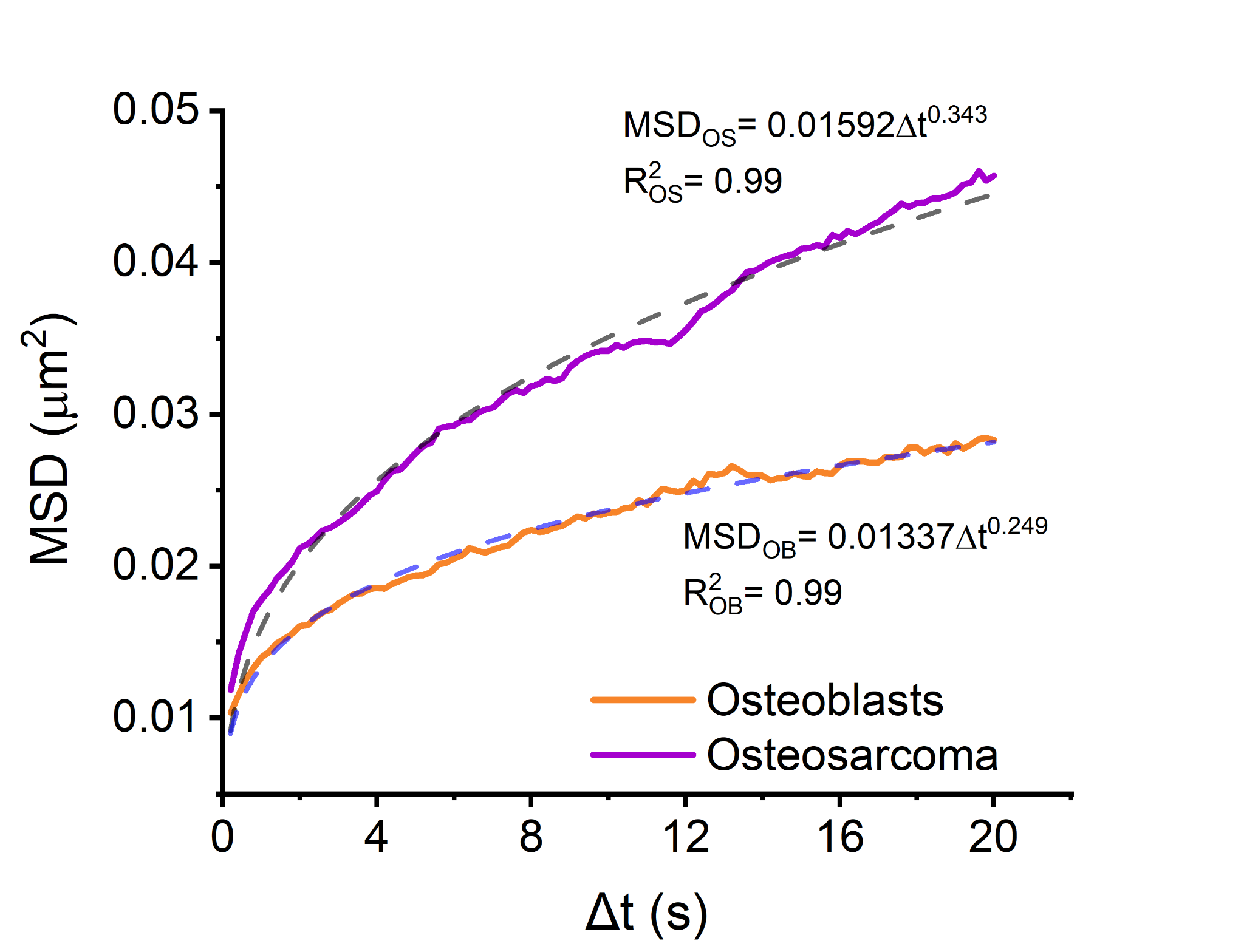
**

**Figure S6. Power-law MSD curve of locus dynamics.** MSD curves of L24 genomic loci (Number of frames = 100, n = 28 trajectories for osteosarcoma, 24 trajectories for normal osteoblasts, N_cell_ ≥ 21) in normal osteoblasts (purple) and osteosarcoma (orange) cells.


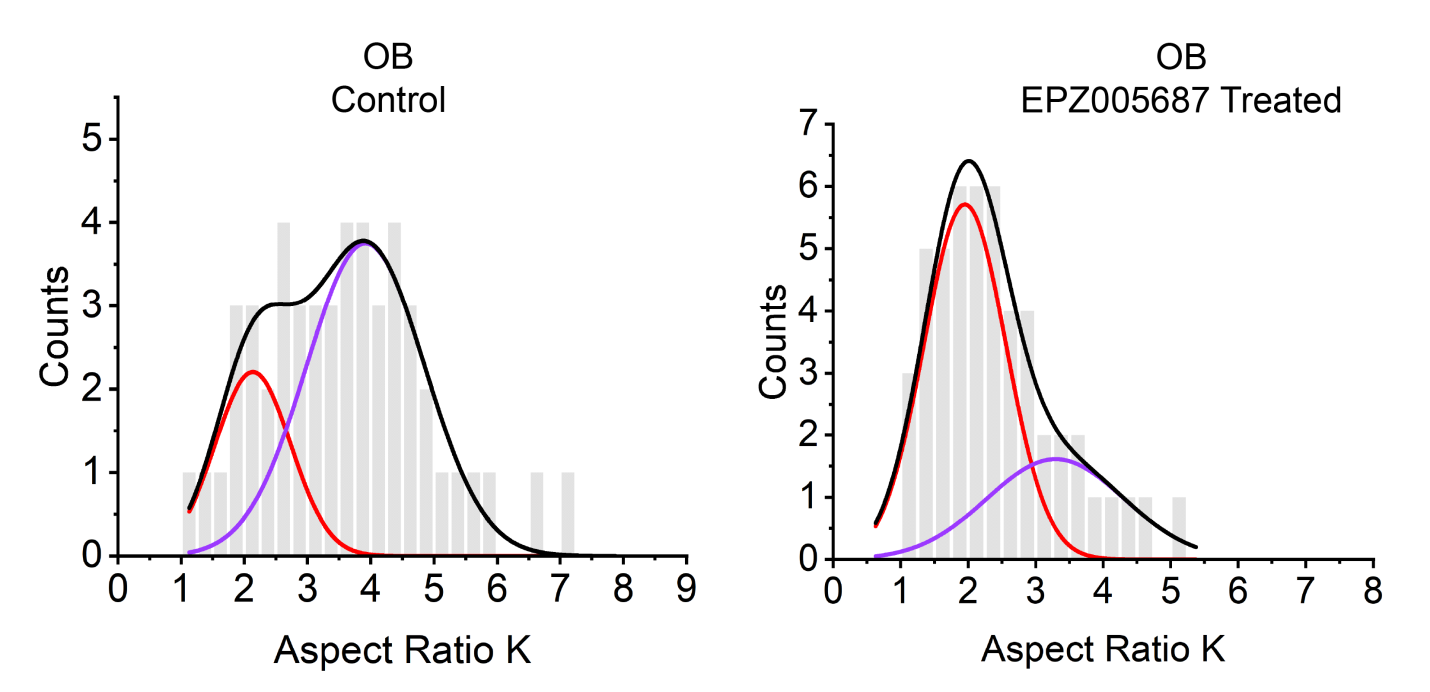


**Figure S7. Distributions of aspect ratio K in OB cells with and without EPZ005687 treatment.** Gaussian bimodal fitting of aspect ratio (K) distribution of C19q conformations for osteoblasts (OB) cells under different EPZ005687 treatment conditions: (A) Control (OB Control, N_C19q_= 50) (B) EPZ005687 treatment (OB EPZ005687 treated, N_C19q_=50). The black line represents the overall bimodal Gaussian fit. Purple and green lines represent the individual Gaussian distributions corresponding to collapsed and extended conformations, respectively.


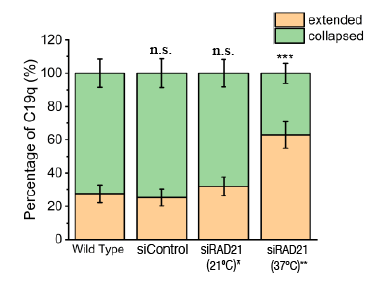


**Figure S8. C19q chromosome conformations are sensitive to cell survival conditions.** Chromosome conformations changed based on exposure to different conditions in RAD21 knockdown OS cells. Percentages of cells with collapsed (green) and extended (orange) C19q conformations in osteosarcoma cells under indicated conditions (NC19q= 110 for each condition). Wild Type (no treatment) and siControl cells were imaged under the same condition as siRAD21 (37℃ with humidity and CO_2_ supplies.), whereas siRAD21 was imaged under room temperature (37℃) without humidity and CO_2_ supplies. The significance test was calculated by using Fisher’s exact test. *, p<0.05; **p<0.01, ***p<0.001; n.s., non-significant.

**Movie S1.** This movie shows a typical movement of the C19q in the osteoblast (hFob1.19) cell nucleus recorded over 70 minutes (each 2D image is a projection from 10 z-slices). The C19q is labeled by GFP, and a false green color was applied to enhance visualization. The movie plays at 3 Hz. Individual frames from this movie are shown in Figure 1F.

**Movie S2.** This movie shows a typical movement of the C19q in the osteosarcoma (U2OS) cell nucleus recorded over 70 minutes (each 2D image is a projection from 10 z-slices). The C19q is labeled by GFP, and a false green color was applied to enhance visualization. The movie plays at 3 Hz. Individual frames from this movie are shown in Figure 1G.

**Table S1: Key resource**

| **Reagent or Resource** | **Source** | **Identifier** |
| --- | --- | --- |
| **Antibodies** | | |
| Rabbit polyclonal anti-CTCF Antibody | Cell Signaling Technology | CAT#: 2899S,  RRID: AB_2086794 |
| Rabbit polyclonal anti-H2B-V119 Antibody | Cell Signaling Technology | CAT#: 8135S,  RRID: AB_10891053 |
| Rabbit polyclonal anti-H3 Antibody | Cell Signaling Technology | CAT#: 9715S,  RRID: AB_331563 |
| Rabbit monoclonal anti-H2A-D603A Antibody | Cell Signaling Technology | CAT#: 12349S,  RRID: AB_2687875 |
| Rabbit polyclonal anti-RAD21 Antibody | Proteintech | CAT#: 27071-1-AP,RRID: AB_2880742 |
| Rabbit polyclonal anti-GAPDH Antibody | Proteintech | CAT#: 10494-1-AP,RRID: AB_2263076 |
| Rabbit polyclonal anti-α-Tubulin Antibody | Proteintech | CAT#: 11224-1-AP,  RRID: AB_2210206 |
| Rabbit polyclonal anti- H3K9me2/3 Antibody | Abcam | CAT#: ab8898,  RRID: AB_306848 |
| Rabbit polyclonal anti- H3K27me3 Antibody | Active motif | CAT#: 39155,  RRID: AB_2561020 |
| Rabbit monoclonal anti – H3K4me2 | Millipore | CAT#: 07-030,  RRID: AB_310342 |
| Anti-Rabbit antibody | Thermo Fisher Scientific | CAT#: 81-1620,  RRID: AB_2534006 |
| **Chemicals, peptides, and recombinant proteins** | | |
| Formaldehyde | Thermo-scientific | CAT#: 28906 |
| TransIT-X2 | Mirus | CAT#: MIR 6003 |
| Lenti-X concentrator | Takara | CAT#: 631231, RRID: |
| T4 DNA Ligase | NEB | CAT#: M0202L |
| RNAse A | Thermo Scientific | CAT#: EN0531 |
| Klenow exo | NEB | CAT#: M0212L |
| Dynabeads Protein A | Invitrogen | CAT#: 10001D |
| SYBR green master mix | Biorad | CAT#: 1725270 |
| cOmplete Protease inhibitor Cocktail | Sigma | CAT#: 11697498001 |
| Proteinase K | Zymo research | CAT#: D3001-2-20 |
| PMSF | Roche | CAT#: 10837091001 |
| Urea | Research Product International | CAT#: U20200-100.0 |
| Triton-X 100 | Sigma | CAT#: T9284-500ML |
| NaN_3_ | Ricca | CAT#: 7144.8-16 |
| NaCl | Fisher Chemical | CAT#: S271-1 |
| EDTA | Sigma Aldrich | CAT#: E9884-100G |
| EGTA | Research Product International | CAT#: E14100-50.0 |
| HEPES pH 8.0 | Sigma | CAT#: H0887-100ML |
| IGEPAL CA-630 | Sigma Aldrich | CAT#: I3021-50ML |
| DRB | Sigma Aldrich | CAT# : D1916-10MG |
| EPZ005687 | Selleckchem.com | CAT# : S7004 |
| DMSO | Sigma Aldrich | CAT# : D8418-100ML |
| **Critical commercial assays** | | |
| End-It DNA End-Repair Kit | Lucigen | CAT#: ER81050 |
| Click-iT RNA Alexa Fluor 594 Imaging Kit | Thermo Scientific | CAT#: C10330 |
| Qubit Protein Assay kit | Invitrogen | CAT#: Q-33211 |
| MinElute PCR purification kit | Qiagen | CAT#: 28004 |
| Mycoalert PLUS kit | Lonza | CAT#: LT07-703 |
| **Deposited data** | | |
| Public RNA seq | https://doi.org/10.18632/oncotarget.26029 | GEO: GSE118488 |
| Public H3K27ac U2OS | https://doi.org/10.1038/s41592-021-01224-1 | GEO: GSE139190 |
| Public H3K27ac hFOB 1.19 | https:// [10.1038/s41418-022-01031-x](https://doi.org/10.1038/s41418-022-01031-x) | GEO: GSE152921 |
| Public RNA-seq of U2OS with RAD21 knockdown | https://www.nature.com/articles/cddis2017345 | GEO: GSE89799 |
| **Experimental models: Cell lines** | | |
| U-2-OS | ATCC | CAT#: HTB-96,  RRID: CVCL_0042 |
| hFOB 1.19 | ATCC | CAT#: CRL-11372,  RRID: CVCL_3708 |
| HEK 293T | ATCC | CAT#: CRL – 3216,  RRID: CVCL_0063 |
| Mouse C2C12 myoblasts | Laboratory of Dr. Benjamin Stanton (NCH) | CAT#: CRL-1772,RRID: CVCL_0188 |
| **Oligonucleotides** | | |
| Negative Control #1 | Ambion | CAT#: 4390843 |
| CTCF siRNA | Ambion | CAT#: 4392420, ID: s20966 |
| RAD21 siRNA | Ambion | CAT#: 4392420, ID: s531212 |
| Primer: HOXB7 Forward –  GAGTAACTTCCGGATCTACCC | IDT | N/A |
| Primer: HOXB7 Reverse –  CGTCAGGTAGCGATTGTAGTG | IDT | N/A |
| Primer: GAPDH Forward –  ACATCGCTCAGACACCATG | IDT | N/A |
| Primer: GAPDH Reverse –  TGTAGTTGAGGTCAATGAAGGG | IDT | N/A |
| Illumina Adaptors and Multiplex Primers | IDT | Kidder and Zhao, 2014 |
| Recombinant DNA | | |
| pPUR-hU6-Sirius-8XMS2-mU6-Sirus-8XPP7 | Addgene | CAT#: 121944  RRID: Addgene_121944 |
| pCMV-dR8.2 dvpr | Addgene | CAT#: 8455  RRID: Addgene_8455 |
| pCMV-VSV-G | Addgene | CAT#: 8454  RRID: Addgene_8454 |
| **Software and algorithms** | | |
| FastQC (version 0.72) | Andrews, S., 2010. | https://www.bioinformatics.babraham.ac.uk/projects/fastqc/ |
| HISAT2 (version 2.1.0) | https://doi.org/10.1038/nmeth.3317 | <http://www.ccb.jhu.edu/software/hisat/> |
| FeatureCounts (version 1.6.4) | <https://doi.org/10.1093/bioinformatics/btt656> | [http://subread.sourceforge.net](http://subread.sourceforge.net/) |
| DESeq (version 1.34.0) | https://doi.org/10.1186/s13059-014-0550-8 | <http://www.bioconductor.org/packages/release/bioc/html/DESeq2.html> |
| TxDb.Hsapiens. UCSC.hg38.knownGene | Team B.C. and Maintainer B.P., 2019 | <https://bioconductor.org/packages/TxDb.Hsapiens.UCSC.hg38.knownGene/> |
| GenomicRanges | <https://doi.org/10.1371/journal.pcbi.1003118> | https://bioconductor.org/packages/release/bioc/html/GenomicRanges.html |
| R (version 4.0.5) | CRAN | https://cran.r-project.org |
| Mathematica (version 13.3) | Wolfram | https://www.wolfram.com/mathematica/ |
| ChIP-seq Pipeline 2 (version 1.3.6) | ENCODE Consortium | <https://github.com/ENCODE-DCC/chip-seq-pipeline2> |
| Olympus cellSens Software | Olympus cellSens Software | <http://www.olympus-lifescience.com/en/software/cellsens/>  (RRID:SCR_014551) |
| ImageJ | Abràmoff, Michael D., Paulo J. Magalhães, and Sunanda J. Ram, 2004 | <http://wsr.imagej.net/distros/> |
| OriginLab | OriginPro, Version 2023. OriginLab Corporation, Northampton, MA, USA. | https://www.originlab.com |
| **Other** | | |
| T4 DNA ligase buffer (10x) | NEB | CAT# : B0202A |
| E-Gel EX agarose gel, 2% | ThermoFisher | CAT# : G401002 |
| Bolt Bis-Tris protein gels, 8% | ThermoFisher | CAT# : NW00082BOX |
| Bolt Bis-Tris protein gels, 12% | ThermoFisher | CAT# : NW00122BOX |
| MOPS buffer | ThermoFisher | CAT# : B0001 |
| MES buffer | ThermoFisher | CAT# : B0002 |
| Tris-Cl | ThermoFisher | CAT# : J22638-AP |
| TBS buffer, pH 7.5 | Quality Biological | CAT# : 351-086-101 |
| 0.45µm polyvinylidene fluoride filter | Fisher Scientific | CAT# : 09-719D |
| RIPA buffer (10x) | ThermoFisher | CAT# : 9806S |
| Covaris milliTUBE 1ml AFA Fiber | Covaris | CAT# : 520135 |
| Vybrant DyeCycle Ruby Stain | Invitrogen | CAT# : V10309 |
| Block-IT RNA | Life technology | CAT# : BT00061 |
