# Supplementary material for: Differential regulation of mesoscale chromosome conformations in osteoblasts and osteosarcoma
Source: Genome Biol. 2025 Sep 26;26:307. doi: 10.1186/s13059-025-03785-2 (PMC12465978; doi:10.1186/s13059-025-03785-2)
Supplement: Supplementary file 2 — Additional file 2. Uncropped western blot images and RT-PCR gel image. [file 13059_2025_3785_MOESM2_ESM.pptx]

## Slide 1
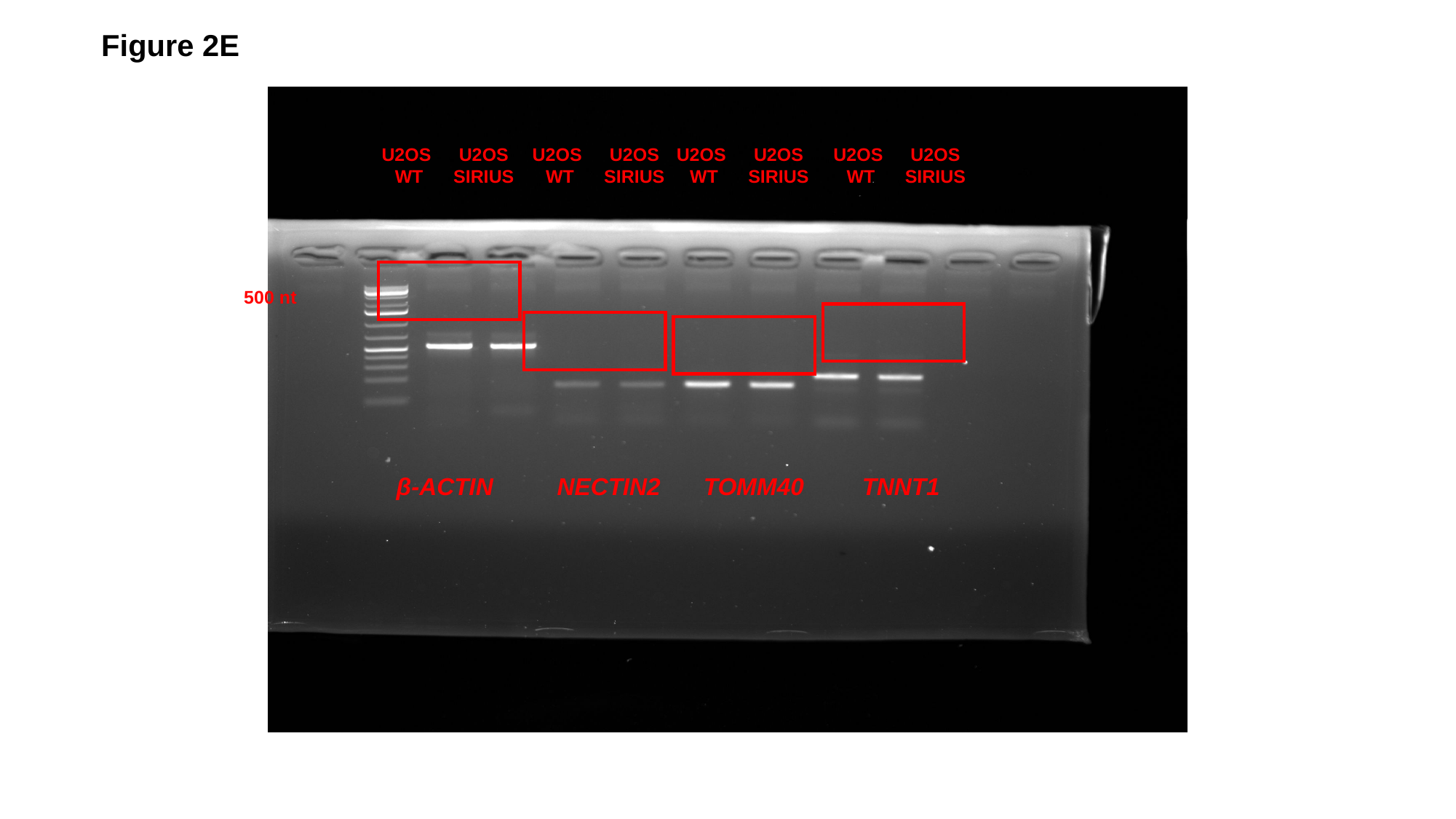

Figure 2E
U2OS
WT
U2OS
SIRIUS
U2OS
WT
U2OS
SIRIUS
U2OS
WT
U2OS
SIRIUS
U2OS
WT
U2OS
SIRIUS
500 nt
β-ACTIN
NECTIN2
TOMM40
TNNT1

## Slide 2
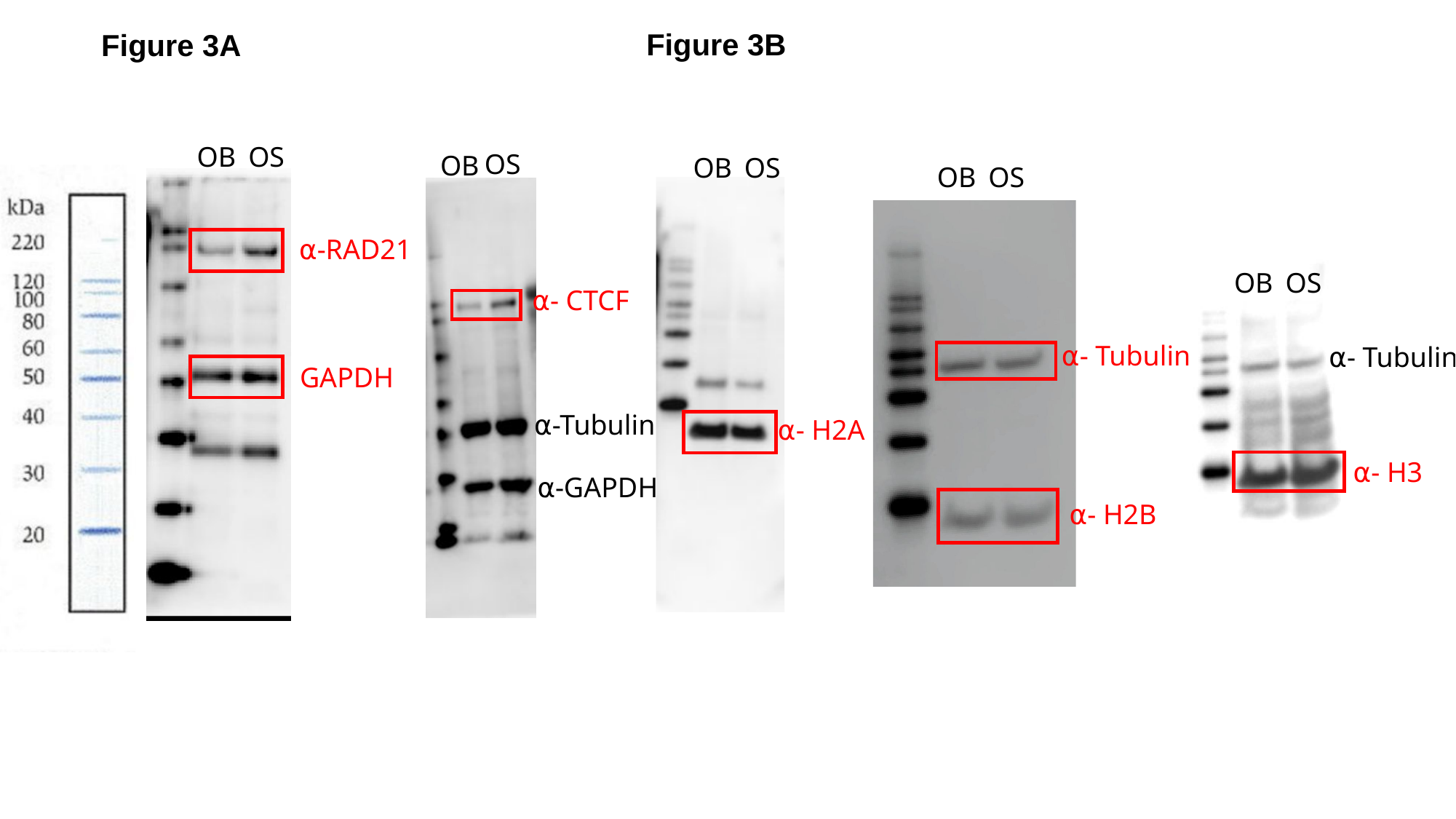

Figure 3B
Figure 3A
OB
OS
⍺-RAD21
GAPDH
OS
OB
OB
OS
OB
OS
OB
OS
⍺- CTCF
⍺- Tubulin
⍺- Tubulin
⍺-Tubulin
⍺- H2A
⍺- H3
⍺-GAPDH
⍺- H2B

## Slide 3
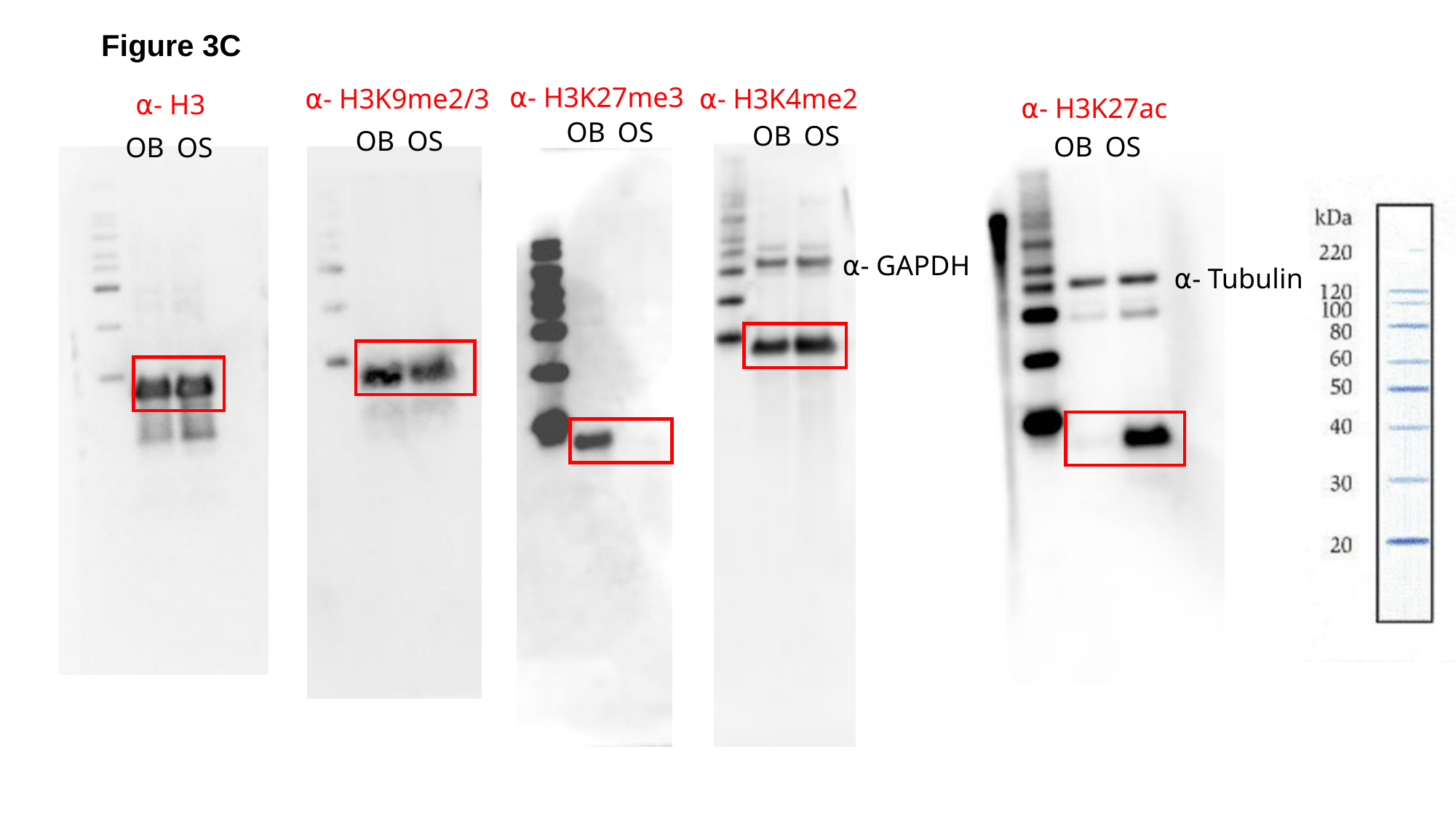

Figure 3C
⍺- H3K27me3
⍺- H3K9me2/3
⍺- H3K4me2
OB
OS
⍺- GAPDH
⍺- H3
⍺- H3K27ac
OB
OS
⍺- Tubulin
OB
OS
OB
OS
OB
OS

## Slide 4
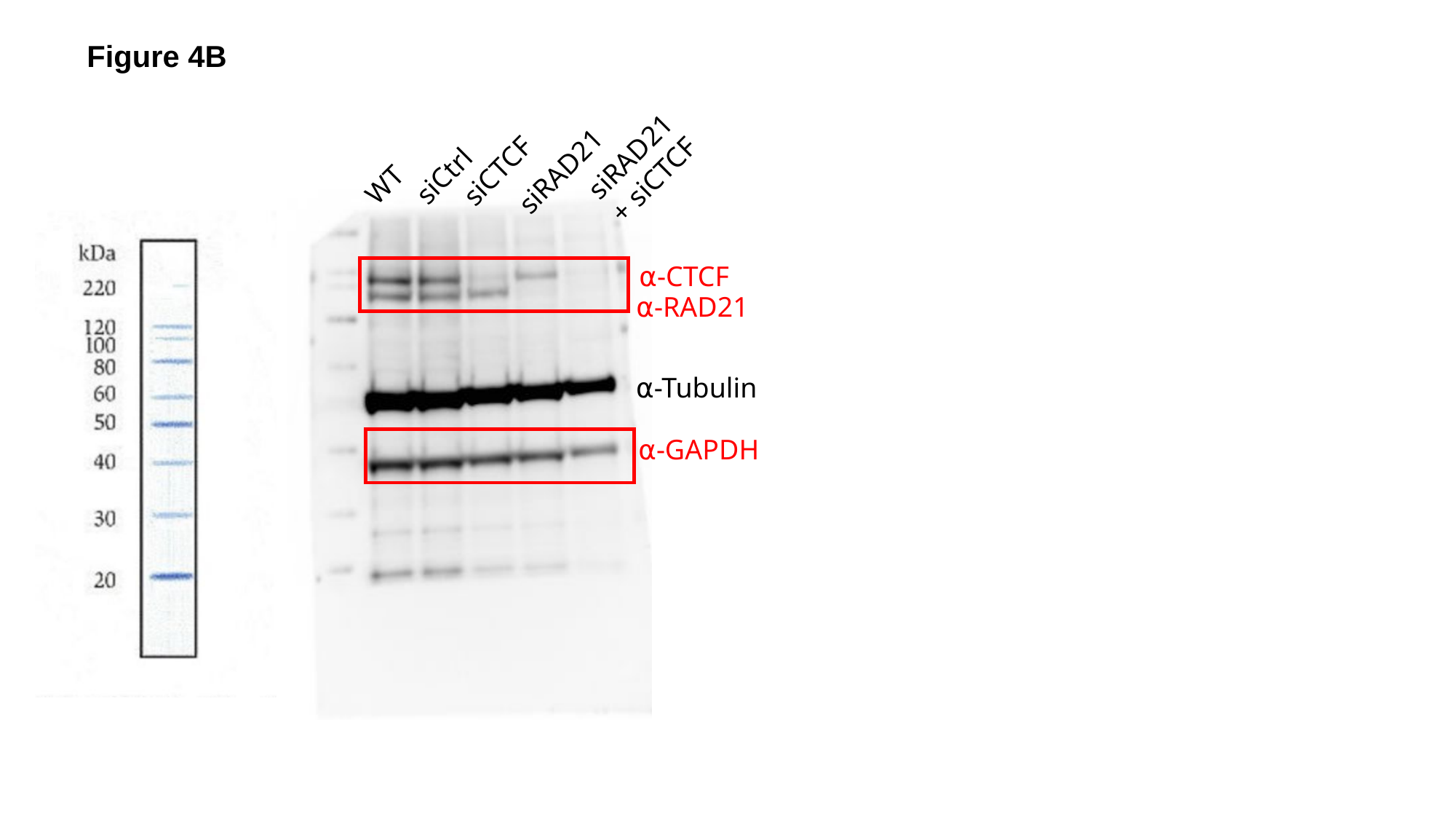

Figure 4B
siRAD21 + siCTCF
siCTCF
siRAD21
siCtrl
WT
⍺-CTCF
⍺-RAD21
⍺-Tubulin
⍺-GAPDH

## Slide 5
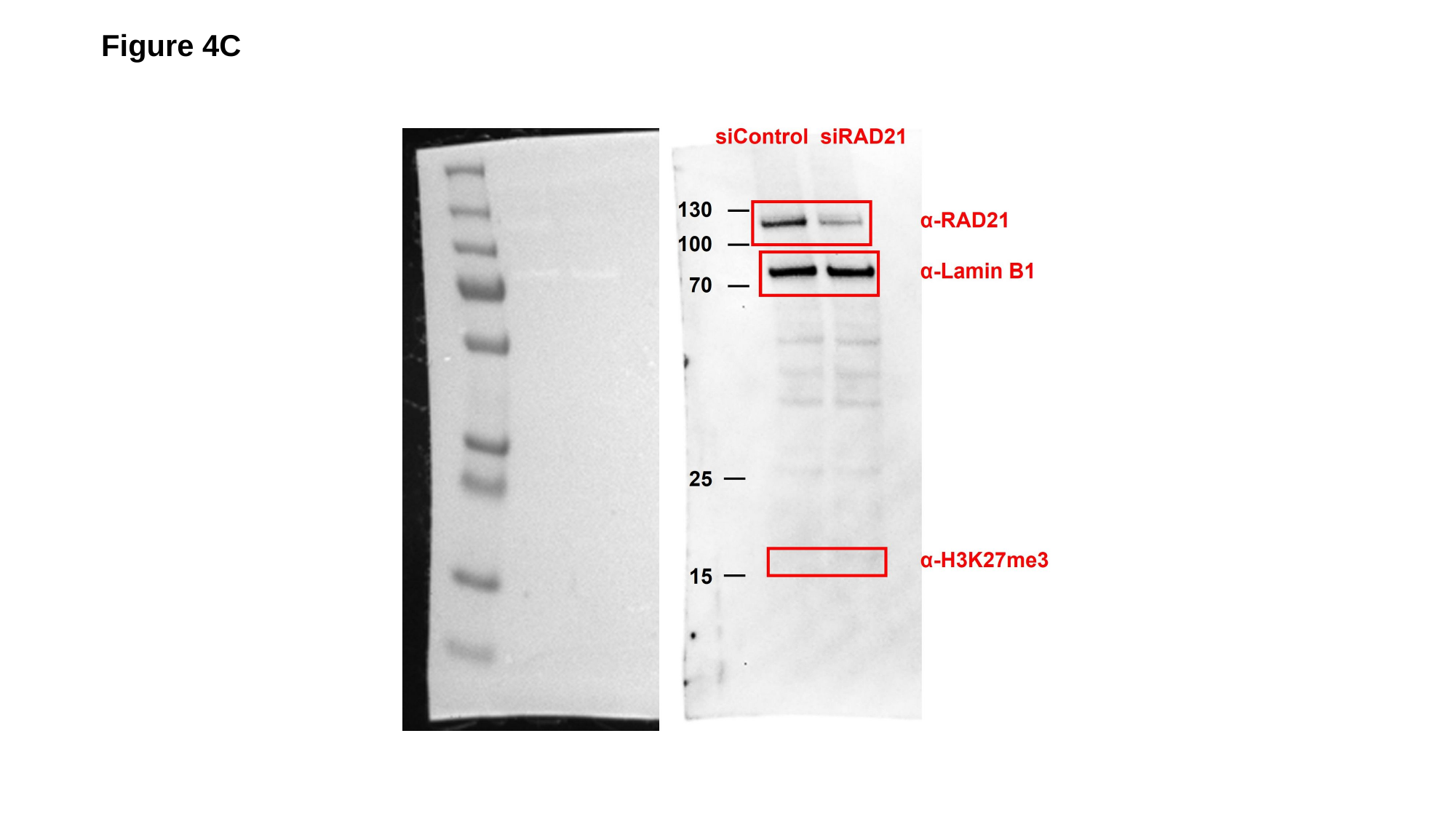

Figure 4C

## Slide 6
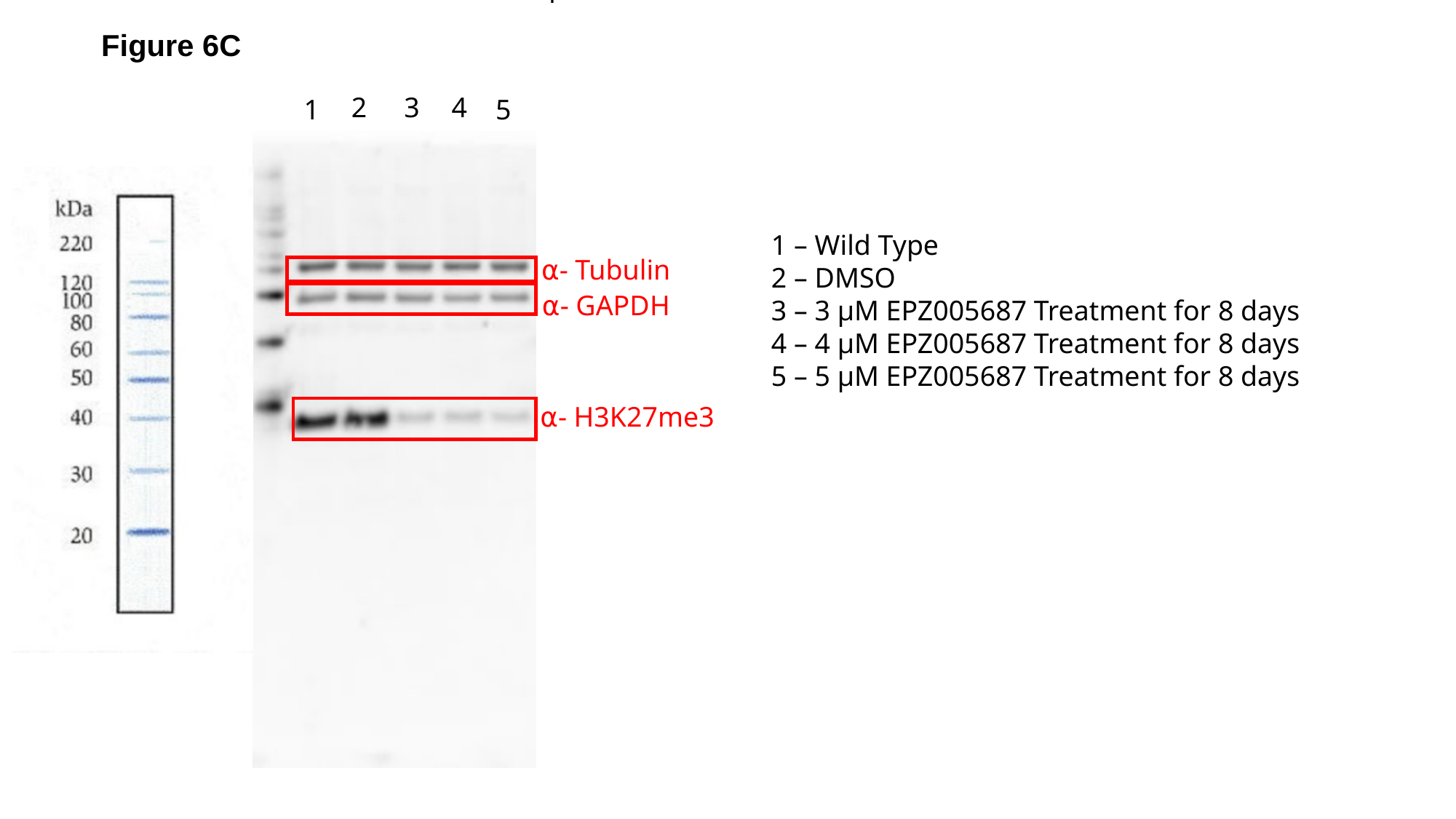

1
Figure 6C
4
2
3
1
5
⍺- Tubulin
⍺- GAPDH
⍺- H3K27me3
1 – Wild Type
2 – DMSO
3 – 3 µM EPZ005687 Treatment for 8 days
4 – 4 µM EPZ005687 Treatment for 8 days
5 – 5 µM EPZ005687 Treatment for 8 days

## Slide 7
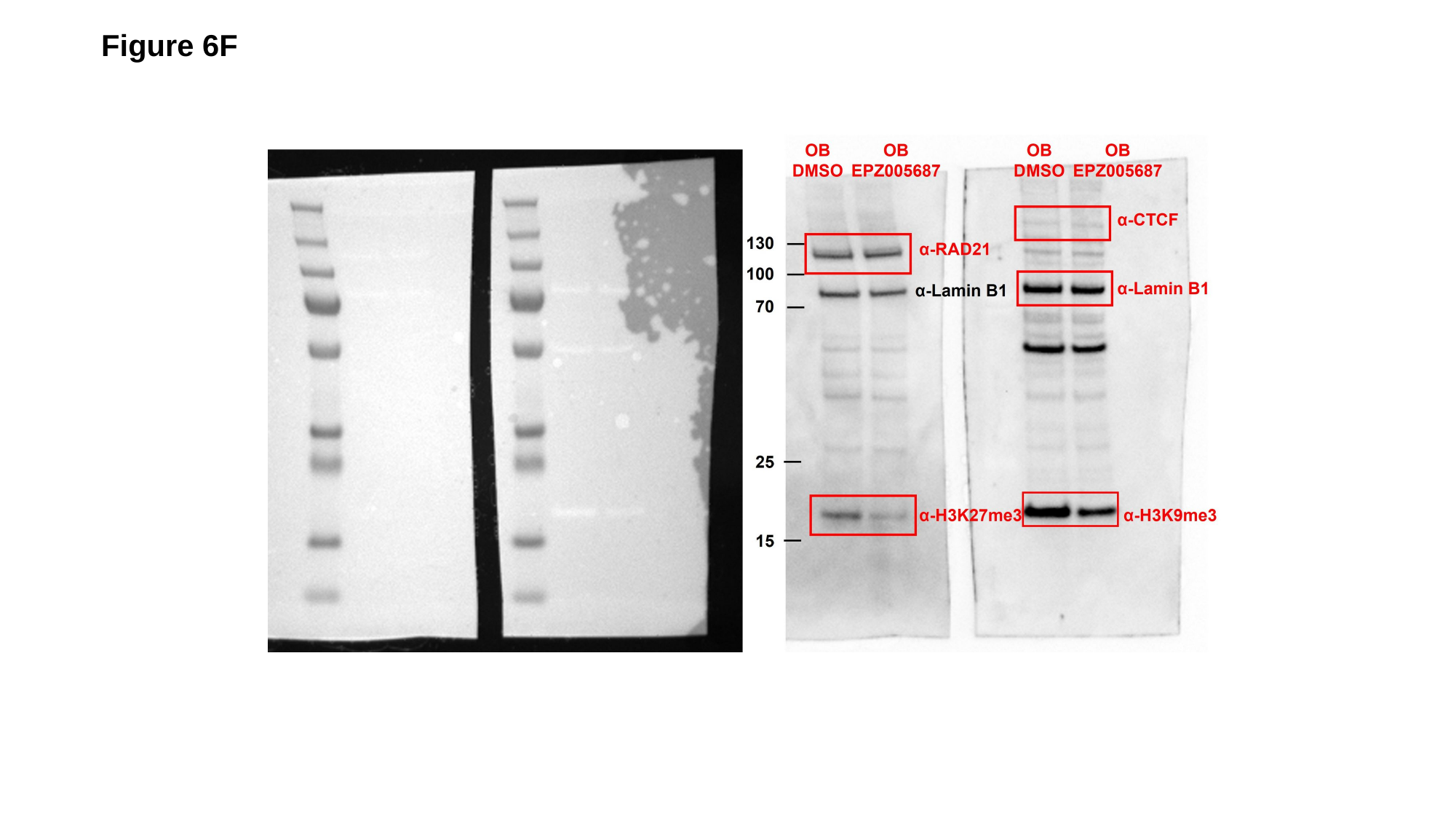

Figure 6F
